# Drug Repurposing in Parasitology: New Uses for Old Drugs to Be Used Through the Host–Parasite Interface

**DOI:** 10.3390/pathogens15090900

**Published:** 2026-08-26

**Authors:** Víctor Hugo Del Río-Araiza, Melodía Rubí Castro-Pérez, Romel Hernández-Bello, Lilia Díaz, Jorge Morales-Montor

**Affiliations:** 1 Departamento de Parasitología, Facultad de Medicina Veterinaria y Zootecnia, Universidad Nacional Autónoma de México, Ciudad de México 04510, Mexico; melodiarubi@comunidad.unam.mx; 2 Departamento de Microbiología, Facultad de Medicina, Universidad Autónoma de Nuevo León, Monterrey 64460, Mexico; romel.hernandezbl@uanl.edu.mx; 3 Departamento de Inmunología, Instituto de Investigaciones Biomédicas, Universidad Nacional Autónoma de México, Ciudad de México 04510, Mexico; lili.dize@hotmail.com

**Keywords:** old compounds, parasitology, antiparasitic drugs

## Abstract

Drug repurposing in parasitology gives existing medications new applications, such as using antibiotics for malaria, antimalarials for worms, and antifungals/cancer drugs for protozoa. This strategy saves time and money by passing early-stage safety testing for established human compounds. Although advances in genomics and proteomics have greatly expanded our understanding of parasite biology and facilitated the identification of potential therapeutic targets, they have translated into relatively few new antiparasitic drugs. This limitation highlights the need for alternative strategies such as drug repurposing. The basic biology of the host–parasite interface has not been thoroughly investigated despite its importance. The multifaceted immune–endocrine interplay may also help clarify the frequently inconsistent observations associated with host age and sex during infections, while providing insights that extend beyond current parasite management approaches. This review summarizes the potential uses and applications of old drugs as antiparasites, including, for example, the use of hormone agonists and antagonists and hormone analogues, while also discussing other drugs that have been isolated from natural products that are not used in the field.

## 1. Introduction

Parasitic diseases remain a major cause of global morbidity and mortality, yet the development of new antiparasitic drugs has progressed slowly because of the high costs, lengthy development timelines, and limited commercial incentives associated with drug discovery. Although compounds such as nitazoxanide have expanded the therapeutic options available against both protozoan and helminth parasites, the emergence of drug resistance and the limited introduction of new drug classes continue to compromise parasite control [1,2]. These challenges have stimulated increasing interest in alternative therapeutic strategies, particularly drug repurposing, which seeks to identify new antiparasitic applications for clinically approved compounds while reducing development time, costs, and the risks associated with de novo drug discovery [3].

Among these strategies, the study of host–parasite interactions has provided new insights into the mechanisms that regulate parasite establishment, development, and survival. In particular, hormones and their antagonists have emerged as promising therapeutic tools because they can interfere with parasite colonization, differentiation, and reproduction, while simultaneously modulating host immune responses [4]. Advances in genomics and molecular parasitology have facilitated the identification of parasite molecules regulated by endocrine signals, enabling the evaluation of compounds such as tamoxifen, RU-486, fadrozole, and flutamide as potential antiparasitic agents [5]. Furthermore, a better understanding of the neuroimmunoendocrine interactions occurring during host–parasite relationship may contribute to the development of selective hormonal analogues capable of disrupting parasite biology [6]. In parallel, animal models and complementary in vitro systems continue to play a fundamental role in evaluating the effects of these compounds on parasite growth, reproduction, and viability, while providing experimental evidence for their potential therapeutic application [7].

Growing interest has also focused on the repurposing of approved drugs originally developed for oncology and other proliferative disorders. Because parasite proliferation and reproduction are fundamental determinants of infection outcome, antiproliferative compounds may also interfere with parasite development and survival [8]. Likewise, increasing knowledge of parasite genomes and transcriptomes has improved the identification of molecular targets involved in parasite colonization and reproduction, facilitating the search for new therapeutic applications of existing drugs [8]. Hormonal regulation has also been shown to influence parasite development. For example, progesterone promotes scolex evagination and developmental processes in *Taenia solium* cysticerci [9]. Recent studies have identified a membrane progesterone receptor component (PGRMC) in *Taenia solium*, providing the first molecular evidence that host progesterone may directly regulate parasite physiology through receptor-mediated signaling [10]. Despite these advances, the discovery of new antiparasitic drugs remains challenging because of the high costs, complex screening procedures, and high attrition rates associated with conventional drug development [11].

These limitations are further compounded by the widespread emergence of drug resistance. Antiparasitic resistance has been reported across all major classes of anthelmintics and has become a major challenge for both veterinary and human medicine [12,13,14]. Although improved sanitation and livestock management practices can reduce parasite transmission, chemotherapy remains the cornerstone of parasite control, relying on a relatively limited number of drug classes, including benzimidazoles, macrocyclic lactones, imidazothiazoles, and praziquantel [12,15]. Consequently, identifying new therapeutic applications for clinically approved drugs represents an attractive strategy to accelerate the development of alternative antiparasitic interventions.

In this review, we summarize current advances in drug repurposing for parasitic diseases, with particular emphasis on compounds that target pathways operating at the host–parasite interface, including hormones, hormone antagonists, antiproliferative agents, and other approved drugs that have shown promising antiparasitic activity.

## 2. Neuroimmunoendocrine Regulation and Parasite Signaling Pathways as Targets for Drug Repurposing

The host–parasite relationship constitutes a dynamic biological interface in which endocrine, immune, and neural signals can influence parasite establishment, development, reproduction, and survival [16,17,18]. Rather than acting as independent systems, the nervous, endocrine, and immune networks communicate through hormones, neurotransmitters, cytokines, and other soluble mediators [16]. During parasitic infections, these signals may modify host susceptibility and immune responses while simultaneously acting directly on parasites capable of recognizing or responding to host-derived molecules [17,19]. Consequently, the neuroimmunoendocrine network represents not only a determinant of infection outcome but also a potential source of pharmacological targets for drug repurposing [16,17,18].

Sex steroids represent one of the best-characterized examples of this bidirectional communication. Estrogens, progesterone, and androgens can modify innate and adaptive immune responses in the host, thereby indirectly affecting parasite establishment and persistence [17]. However, their effects are not restricted to host immunomodulation. Several parasites respond directly to host-derived steroid hormones, suggesting the existence of parasite molecules capable of recognizing endocrine signals [10,17,20]. In *Taenia solium*, for example, progesterone promotes scolex evagination and parasite development [21], and PGRMC has been identified in subtegumental tissues [10]. Molecular modeling indicates that this protein can potentially bind progesterone, estradiol, testosterone, and dihydrotestosterone with different affinities, providing molecular evidence for direct endocrine communication at the host–parasite interface [10].

Recognition of host-derived signals must ultimately be translated into intracellular responses within the parasite. These responses involve signaling networks controlling processes such as Ca^2+^ homeostasis, cyclic nucleotide signaling, protein phosphorylation, mitochondrial function, redox balance, lipid metabolism, and gene expression [22,23,24,25]. Although the relative importance of these pathways varies among parasite taxa and developmental stages, their disruption can compromise essential biological processes, including motility, differentiation, invasion, proliferation, and survival [24,26]. Thus, parasite signaling pathways provide a mechanistic link between extracellular signals at the host–parasite interface and the cellular effects produced by repurposed drugs [23].

Ca^2+^ signaling is particularly relevant in protozoan parasites because intracellular Ca^2+^ regulates processes such as motility, differentiation, host–cell invasion, and parasite survival [27]. In trypanosomatids, Ca^2+^ homeostasis depends on specialized intracellular stores and organelles, including the endoplasmic reticulum, mitochondrion, and acidocalcisomes [28,29]. Pharmacological disruption of these systems can therefore produce profound effects on parasite viability. This provides the mechanistic basis for repurposed compounds such as amiodarone, whose trypanocidal activity has been associated with disruption of intracellular Ca^2+^ homeostasis and mitochondrial function [29].

From a drug repurposing perspective, these interactions provide two complementary opportunities for therapeutic intervention: modulation of host pathways that influence susceptibility or protective immunity, and direct targeting of parasite receptors, signaling molecules, metabolic pathways, or intracellular organelles [30,31,32,33]. Importantly, the same compound may act simultaneously at both levels. Therefore, understanding the molecular dialogue between host and parasite provides a mechanistic framework for interpreting the antiparasitic effects of drugs originally developed for endocrine disorders, cancer, cardiovascular diseases, and microbial infections [30,34]. The following sections discuss representative examples of repurposed compounds and the host- or parasite-associated pathways through which their antiparasitic effects have been proposed to occur.

## 3. Drug Repurposing Through the Host–Parasite Interface

Increasing evidence indicates that host hormones can directly regulate parasite physiology through specific receptor-mediated mechanisms [10,20]. This recognition provides a rationale for the development of hormone-based therapeutic strategies targeting the host–parasite interface. These strategies include: (a) determining which hormones can directly suppress parasite growth, reproduction, or differentiation independently of the host immune system; (b) developing hormone analogues that specifically target parasites while minimizing off-target effects in the host; and (c) optimizing compounds that competitively bind to parasite hormone receptors, thereby interfering with gene expression and other critical cellular mechanisms [35].

Although the pharmaceutical industry allocates approximately 25 million dollars annually to antiparasitic drug development, the introduction of new drugs into the market remains infrequent, often occurring at intervals of about fifteen years. These agents are primarily designed to compromise parasite survival while ensuring host safety and preventing cross-resistance with existing therapies. The development process is both costly and time-consuming, requiring extensive preclinical testing in animal models to evaluate efficacy and toxicity. This process typically takes between 5 and 10 years, contributing to reduced engagement from both industry and medical research sectors. While advances in parasite genomics hold promise for accelerating and reducing the cost of drug discovery, many parasite genomes are still under investigation, and their practical applications are not expected to be fully realized for several more years.

In response to these challenges, several research groups, including our own, have investigated drug repurposing, reprofiling, and reassignment strategies. These approaches involve evaluating the efficacy of approved drugs in vitro and in vivo against parasitic infections, with the aim of identifying new therapeutic applications beyond their original medical indications [36]. Additionally, the urgent demand for effective control measures in developing countries has driven the evaluation of drugs already approved by the Food and Drug Administration (FDA), with the aim of reducing both development time and associated costs.

Considering that parasite reproduction is a key factor in infection dynamics, it is reasonable to propose that drugs with established antiproliferative activity may also impair parasite reproduction. However, a significant challenge remains in identifying new chemical entities that combine safety with strong antiparasitic efficacy [37]. Current evidence indicates that steroids can produce a broad range of effects on the host immune response—either enhancing or suppressing it—while also influencing the viability of both protozoan and metazoan parasites [17]. Consequently, increasing attention is being directed toward the use of sex hormones, their analogues, and other immunomodulatory compounds as alternative strategies for preventing parasitic diseases [17,37].

These observations have encouraged the evaluation of antihormonal and antiproliferative agents as modulators of parasite gene expression, development, and survival. The following sections summarize the main classes of repurposed compounds that have been investigated using this strategy.

### 3.1. Hormone Modulators

#### 3.1.1. Tamoxifen

Tamoxifen is one of the most widely prescribed drugs for the treatment of estrogen-dependent breast cancer worldwide. As a Selective Estrogen Receptor Modulator (SERM), it acts by blocking estrogen binding in target cells, thereby inhibiting cellular proliferation and tumor progression. It is commonly indicated for both treatment and prevention of breast cancer and is typically administered over extended periods, often for up to five years at daily doses ranging from 20 to 40 mg [38].

Beyond oncology, tamoxifen has been explored for its potential use in parasitic infections. In *Taenia crassiceps*, administration of tamoxifen resulted in a significant reduction in parasite burden—approximately 80% in female mice and 50% in males [39]. This protective effect was associated with increased mRNA expression of IL-2, a cytokine linked to protective immune responses, while IL-4 levels were also elevated but without a clear impact on infection outcomes. In vitro, tamoxifen reduced parasite motility and reproductive capacity. These findings suggest that tamoxifen may act at multiple levels of the host–parasite interaction, enhancing host immune responses while directly impairing parasite survival and replication, thus representing a potential therapeutic alternative for cysticercosis [39]. The antiparasitic activity of tamoxifen is likely mediated by both host-dependent and parasite-directed mechanisms. Besides enhancing IL-2-associated immune responses, tamoxifen directly impairs parasite motility and budding, suggesting interference with cellular pathways required for parasite proliferation. Since cestodes express estrogen-binding proteins, these observations support the hypothesis that modulation of estrogen-dependent signaling contributes to the reduction in parasite survival and reproductive capacity.

Leishmaniasis remains one of the major neglected tropical diseases, and currently available therapies are limited by toxicity, prolonged treatment schedules, and increasing drug resistance [40,41,42,43]. Current treatment options for leishmaniasis remain limited and often suboptimal. Pentavalent antimonials, such as sodium stibogluconate and meglumine antimoniate, continue to be recommended as first-line therapy for cutaneous forms, typically administered at 20 mg/kg/day for 20–28 days [41]. However, these drugs are associated with significant toxicity, variable efficacy, and the need for parenteral administration. Other agents, including amphotericin B and pentamidine, also present important limitations [43]. In contrast, tamoxifen has a well-established safety profile in humans [44] and has demonstrated promising activity against several *Leishmania* species. It exerts a direct leishmanicidal effect and alters the pH of parasitophorous vacuoles from acidic to neutral, thereby enhancing its activity against intracellular amastigotes. In vitro, concentrations of approximately 10 μM inhibit about 50% of *Leishmania amazonensis* growth and viability [45]. In vivo studies using BALB/c mice infected with *Leishmania amazonensis* showed that tamoxifen treatment significantly reduced lesion size, ulceration, and parasite burden, with no detectable toxicity. Treated animals also exhibited reduced inflammation at the infection site [45]. These findings suggest that tamoxifen may represent an effective antileishmanial agent without requiring extended treatment regimens [44]. Mechanistically, tamoxifen disrupts parasite sphingolipid metabolism, alters membrane organization, and neutralizes the acidic pH of parasitophorous vacuoles, thereby compromising intracellular amastigote survival. These effects appear to be independent of estrogen receptor modulation and are associated with direct leishmanicidal activity rather than solely with immunomodulation [45,46]. Recent studies have further optimized the antileishmanial scaffold of tamoxifen by developing hybrid molecules with submicromolar activity and low cytotoxicity, supporting the continued potential of tamoxifen-derived compounds for antiparasitic drug development [47].

Further mechanistic evidence has demonstrated that tamoxifen interferes with sphingolipid biosynthesis in *Leishmania*. Treatment altered the levels of inositolphosphorylceramide and other membrane lipids and inhibited parasite inositolphosphorylceramide synthase, supporting sphingolipid metabolism as an important parasite-directed target of tamoxifen [48]. The therapeutic potential of tamoxifen has also been explored using topical administration. In experimental cutaneous leishmaniasis, topical tamoxifen reduced lesion development and parasite burden, and its combination with pentavalent antimonial therapy further supported the feasibility of local tamoxifen-based approaches [49].

More recently, tamoxifen has reached clinical evaluation in cutaneous leishmaniasis. A randomized non-inferiority trial investigated a sequential pentamidine–tamoxifen regimen in an area where *Leishmania* (*Viannia*) *guyanensis* predominates. Although the tamoxifen-containing regimen showed clinical activity, it did not demonstrate non-inferiority to the pentamidine regimen, indicating that further optimization of dose, schedule, and combination strategies is required before tamoxifen can be considered for clinical use [50].

In the case of *Schistosoma mansoni*, tamoxifen has also demonstrated activity against this trematode. Experimental studies have shown activity against both immature and adult worms in vitro, while treatment of infected mice reduced worm and egg burdens [51]. Target-fishing approaches further identified parasite metabolic proteins as potential molecular targets, suggesting that the antischistosomal effects of tamoxifen may involve disruption of parasite metabolism in addition to its classical estrogen-modulatory activity [51].

Tamoxifen also shows significant activity against *Trypanosoma cruzi*. Treatment with 10 μM induces approximately 90% lysis of bloodstream trypomastigotes, while concentrations of 50 μM inhibit up to 86% of intracellular amastigote development. These antiparasitic effects are associated with apoptosis-like mechanisms linked to disruption of the parasite’s sphingolipid metabolism [52].

Tamoxifen has demonstrated antiparasitic activity against *Plasmodium falciparum* in vitro. Its proposed mechanism of action involves inhibition of enzymes participating in sphingolipid biosynthesis, including glucosylceramide synthase and sphingomyelin synthase, which are essential for membrane biogenesis during erythrocytic development. However, significant parasite inhibition was observed only at relatively high concentrations, indicating that further studies are needed to evaluate its therapeutic potential and optimize its antiparasitic activity [53].

In contrast to the antiparasitic effects described above, tamoxifen may exert an unfavorable effect during chronic *Toxoplasma gondii* infection. Although available data are limited, studies in murine models have shown that treatment with tamoxifen for 14–28 days in chronically infected animals leads to increased parasite burden and significant histopathological and immunohistochemical alterations in organs such as the brain, liver, kidneys, and uterus [54]. These findings indicate that the effects of tamoxifen are parasite-dependent and cannot be generalized across protozoan infections. The increased parasite burden observed during chronic infection suggests that modulation of estrogen signaling may differentially affect host immune regulation depending on the parasite species and stage of infection, emphasizing the complexity of host–parasite endocrine interactions [54].

Taken together, these contrasting findings indicate that the potential repurposing of tamoxifen as an antiparasitic agent is highly parasite-dependent and should be evaluated according to the specific molecular and endocrine interactions established at each host–parasite interface.

#### 3.1.2. Dehydroepiandrosterone (DHEA)

Previous studies have demonstrated that progesterone exerts a protective effect against *Taenia crassiceps* cysticercosis in gonadectomized mice. In these experiments, both male and female neutered mice treated with progesterone exhibited complete protection against infection, whereas control animals remained susceptible [55].

**Figure 1:**
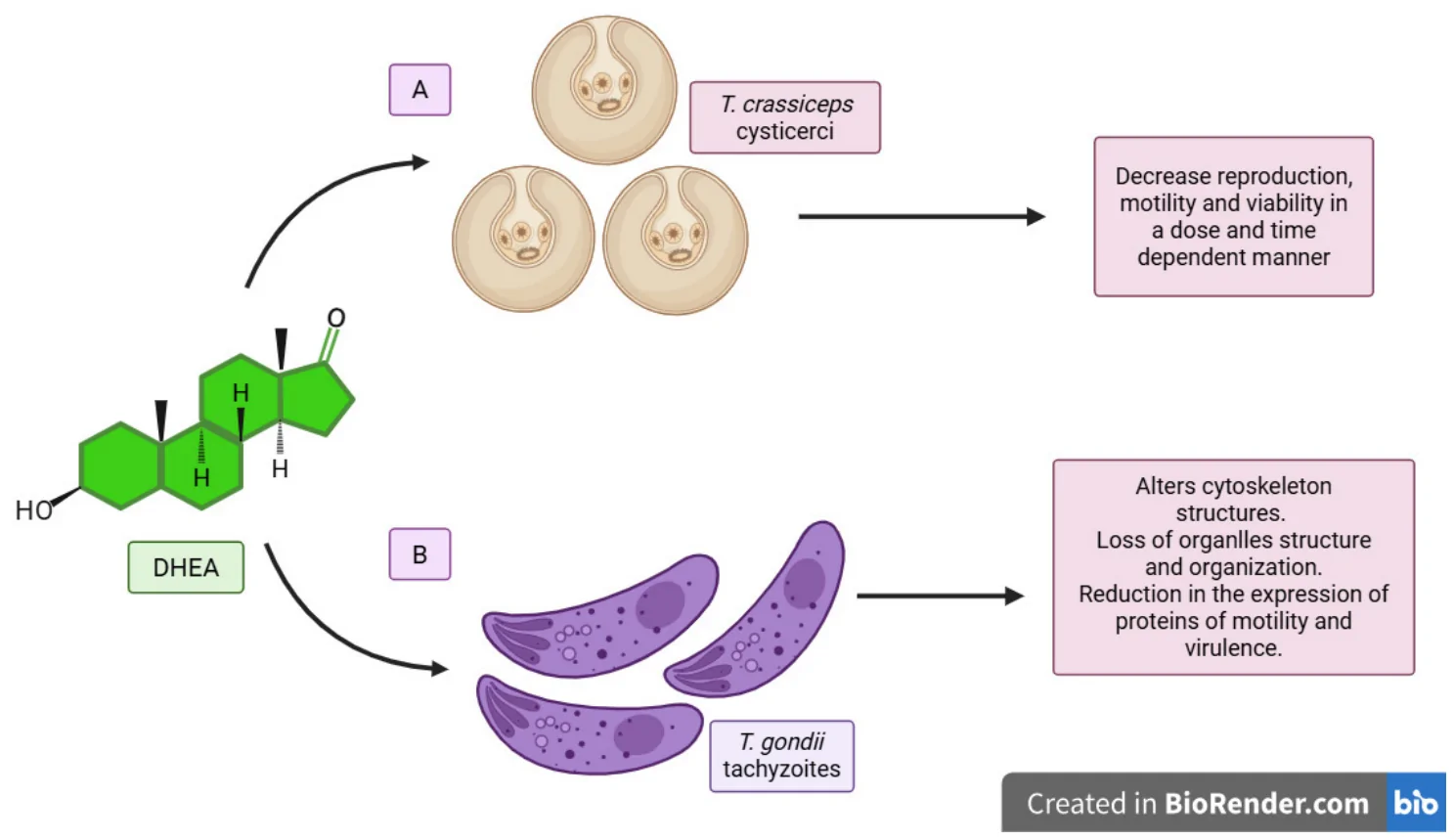
Direct effects of DHEA on parasite physiology. (A) In vitro activity against *Taenia crassiceps* cysticerci. (B) In vitro activity against *Toxoplasma gondii* tachyzoites. Image created with BioRender.

Notably, the level of protection observed surpassed previously reported outcomes, including those achieved through vaccination, while variability in parasite burden among treated animals remained minimal. Further analysis suggested that progesterone was metabolized into DHEA, resulting in elevated DHEA levels rather than sustained progesterone concentrations. This observation supports the hypothesis that the protective effect was mediated by adrenal conversion of progesterone into DHEA. Consistently, administration of DHEA prior to infection reduced parasite burden by approximately 50% relative to untreated controls. Importantly, this effect was not associated with modulation of the host immune response, as no significant changes were observed in mRNA levels of IL-2, IFN-γ, IL-4, or IL-10. In contrast, in vitro exposure of *T. crassiceps* to DHEA resulted in dose- and time-dependent reductions in parasite motility, reproduction, and viability, indicating a direct antiparasitic effect (Figure 1A) [56].

DHEA has been characterized as a potent antiparasitic molecule across multiple experimental systems. Although some studies have reported that exogenous DHEA enhances cellular immunity by increasing the number and activity of natural killer cells [57], these findings do not support an immunomodulatory mechanism, as cytokine expression remained unchanged following treatment [56]. The absence of detectable immune-related effects, despite a marked reduction in parasite burden in vivo and decreased survival in vitro, suggests that DHEA exerts its protective effect primarily through direct action on the parasite. This interpretation is consistent with previously reported effects of DHEA on both metazoan and protozoan parasites [58,59].

In human schistosomiasis, DHEA has been proposed as a contributing factor to the decreased susceptibility observed after puberty [60]. Supporting this hypothesis, treatment of mice with the circulating form DHEA-S provided protection against *Schistosoma mansoni* infection [59]. Additionally, it was observed that declining DHEA levels during the progression of infection in mice are consistent with findings from a *Schistosoma mansoni*–baboon model, in which infected animals exhibited reduced DHEA levels compared to uninfected or re-exposed individuals [61].

Exogenous administration of DHEA has also demonstrated antiparasitic effects in other models. In rats infected with *Trypanosoma cruzi*, DHEA increased the production of lytic antibodies and reduced parasitemia [62]. More recently, the direct trypanocidal activity of DHEA and several steroid derivatives has been evaluated against different *Trypanosoma cruzi* strains. DHEA showed strain-dependent activity, exhibiting greater activity than benznidazole and nifurtimox against epimastigotes of the TH strain, whereas other steroid derivatives showed higher activity against the Ninoa strain. However, DHEA and the evaluated derivatives did not show significant activity against bloodstream trypomastigotes in ex vivo assays. These findings suggest that the antiparasitic activity of DHEA and its derivatives may depend on both parasite strain and developmental stage, and further structural optimization may be required to improve their trypanocidal efficacy [63].

In Syrian golden hamsters experimentally infected with *Cryptosporidium parvum*, prophylactic administration of DHEA before infection significantly reduced fecal oocyst shedding and intestinal parasite colonization. These findings support the potential of DHEA as a prophylactic immunomodulatory strategy rather than as a treatment for established cryptosporidiosis [64]. In *Toxoplasma gondii*, in vitro exposure to DHEA exerted a toxoplasmicidal effect on extracellular tachyzoites by disrupting cytoskeletal organization, leading to loss of cellular structure and decreased expression of proteins involved in motility and virulence (Figure 1B). In vivo, however, DHEA treatment reduced parasite burden in male mice but not in females [65].

These findings further support the concept that androgens may play an important role in limiting *Taenia crassiceps* infection in immunocompetent hosts. Previous studies have suggested that testosterone and DHEA negatively regulate parasite reproduction in both sexes, potentially through modulation of immune responses, including suppression of Th2 pathways and enhancement of Th1 responses, as well as through direct effects on parasite survival, motility, and reproduction [61,66].

Preclinical and clinical studies using the synthetic androstane steroid HE2000 in patients infected with *Plasmodium falciparum* demonstrated approximately a 50% reduction in parasitemia. Reported adverse effects were mild to moderate and transient, supporting the safety, tolerability, and therapeutic potential of this compound when administered via intramuscular or buccal routes [67].

Conversely, inhibition of sex hormones has also been shown to restore protective immune responses in certain contexts. In murine cysticercosis, 17β-estradiol promotes parasite reproduction by suppressing Th1 responses and favoring Th2 polarization [68,69]. Pharmacological inhibition of estrogen synthesis using fadrozole, an aromatase inhibitor that blocks P450 aromatase activity, reduces estradiol production in both males and females. This intervention resulted in a 70% reduction in parasite burden, increased IL-6 serum levels, and a shift from Th2- to Th1-dominated immune responses [70,71], highlighting a potential alternative therapeutic strategy for parasitic infections.

### 3.2. Anticancer Drugs Targeting Parasite Proliferation

#### 3.2.1. Paclitaxel

Paclitaxel (Taxol) is a widely used antineoplastic agent employed in the treatment of various solid tumors, including breast, ovarian, lung, bladder, prostate, and esophageal cancers, as well as melanoma and Kaposi’s sarcoma. Because microtubules are essential components of eukaryotic cells, they have been extensively investigated as drug targets, including in parasitic organisms. In *Leishmania* spp., microtubules represent a promising therapeutic target, and paclitaxel has been evaluated for its antiparasitic potential [72].

Prolonged exposure (>72 h) of *Leishmania major* cultures to high concentrations of paclitaxel (35 mM) significantly reduces parasite viability. Microscopically, treated parasites display rounded morphology, increased size, and markedly reduced motility, supporting a direct deleterious effect of paclitaxel on parasite survival, in addition to indirect effects mediated by macrophage activation [73]. This activity is consistent with paclitaxel’s mechanism of stabilizing microtubules through binding to β-tubulin and inhibiting their depolymerization. Moreover, paclitaxel, administered alone or combined with interferon-gamma (IFN-γ), enhances parasite killing by activated murine macrophages [73]. The antileishmanial potential of paclitaxel has been further investigated both as monotherapy and in combination with established antileishmanial drugs. Paclitaxel was evaluated together with miltefosine and paromomycin, providing additional evidence that taxanes may exert both direct antiparasitic and immunomodulatory effects. These findings support the potential use of paclitaxel-containing combination strategies and further strengthen the rationale for repurposing microtubule-targeting anticancer drugs against *Leishmania* spp. [74].

**Figure 2:**
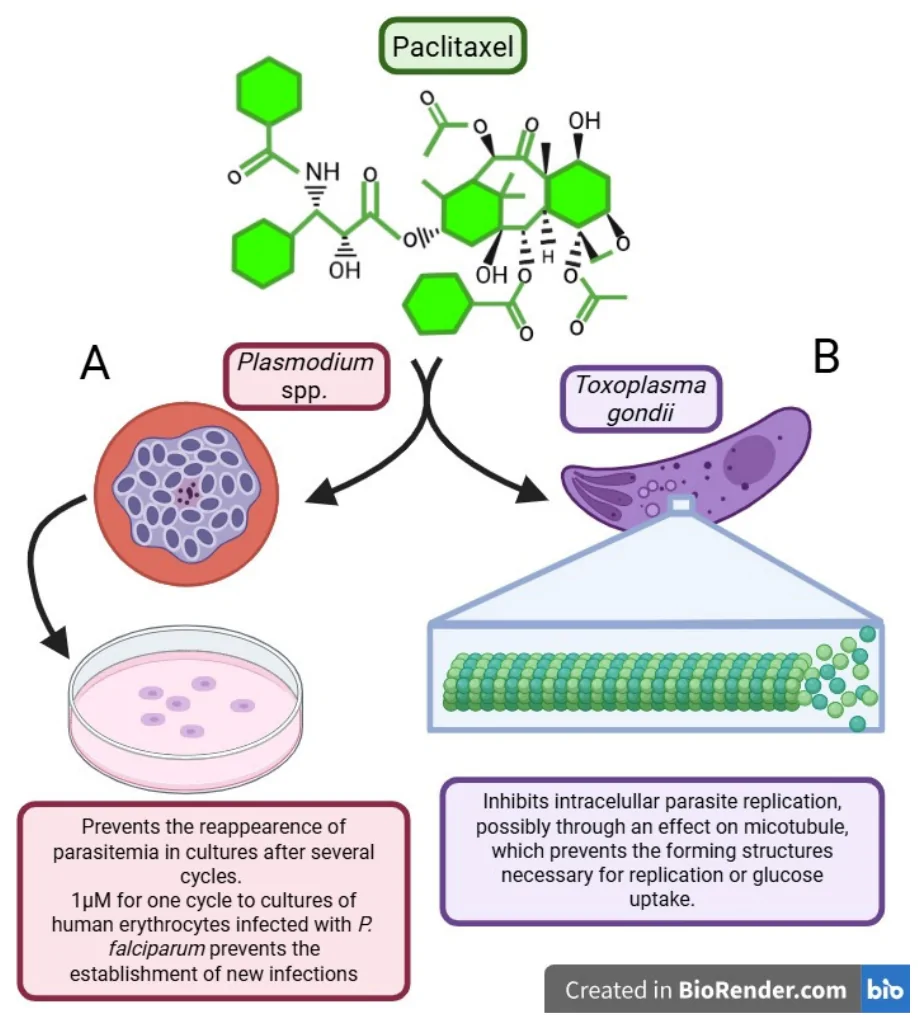
Antiparasitic effect of paclitaxel. (A) Activity against *Plasmodium* spp. (B) Action against *Toxoplasma gondii*. Image created with BioRender.

In malaria research, paclitaxel has shown strong activity against drug-resistant parasites. It effectively eliminates parasitemia in cultures resistant to chloroquine and pyrimethamine [75]. Compared with other antimalarials, paclitaxel demonstrates greater in vitro efficacy by preventing recrudescence after several replication cycles [76,77]. Although it does not appear to directly kill parasites, it rapidly reduces parasite burden, with complete clearance typically achieved within 48 h of treatment initiation [75]. Treatment of *Plasmodium falciparum*-infected erythrocyte cultures with 1 μM paclitaxel prevents the establishment of new infections (Figure 2A). In vivo, a single intraperitoneal dose eliminates parasitemia in mice infected with *Plasmodium chabaudi adami*, and similar effects are observed in *Plasmodium berghei* models, where paclitaxel significantly reduces parasitemia and improves survival rates. Treated animals exhibit slower disease progression and improved survival compared with untreated controls, likely as a result of enhanced eryptosis of infected erythrocytes and their subsequent clearance from the circulation [76].

More recently, the antimalarial potential of paclitaxel has been confirmed against both drug-sensitive and drug-resistant *Plasmodium falciparum* strains. Paclitaxel inhibited intraerythrocytic parasite development at nanomolar concentrations, impaired schizogony and merozoite formation, and reduced gametocyte maturation. Molecular modeling further suggested an interaction with *Plasmodium falciparum* β-tubulin, supporting disruption of parasite microtubule dynamics as a potential mechanism of action. Importantly, paclitaxel showed synergistic activity with dihydroartemisinin against artemisinin-resistant parasites, and this combination markedly reduced parasitemia in experimental models [78].

Paclitaxel also inhibits the intracellular replication of *Toxoplasma gondii*. Treatment of infected fibroblasts with 1 μM paclitaxel for periods ranging from 24 h to several days results in a marked and largely irreversible reduction in parasite replication, which persists after drug withdrawal [77]. Although host cell invasion is not affected, subsequent parasite proliferation is significantly impaired. This inhibitory effect is likely due to disruption of parasite microtubule dynamics, which prevents the formation of structures required for replication and potentially impairs nutrient acquisition such as glucose uptake (Figure 2B). These findings suggest that paclitaxel, already approved for cancer therapy, may have potential as an alternative treatment for *Toxoplasma gondii* infections [77].

In *Trypanosoma cruzi*, paclitaxel inhibits parasite replication under in vitro conditions. At µM concentrations, it disrupts cytokinesis, permitting continued replication of cellular organelles, including the nucleus, kinetoplast, and flagellum, while preventing completion of cell division. This leads to the formation of motile parasites with abnormal morphology and excess organelles [79]. Subsequent studies demonstrated that bloodstream trypomastigotes are more sensitive to paclitaxel than epimastigotes, and that treatment induces pronounced ultrastructural alterations, particularly affecting parasite shape and integrity [80].

#### 3.2.2. Docetaxel

Docetaxel is a chemotherapeutic agent widely used in the treatment of various malignancies, including breast cancer, prostate cancer, and non-small cell lung cancer, and is also approved for several other tumor types. As a microtubule-stabilizing compound, docetaxel (Taxotere) has also been investigated for its effects on parasitic organisms.

In *Plasmodium falciparum*, docetaxel has been shown to interfere with the parasite’s intraerythrocytic development [81]. When applied during the ring stage, the drug induces disruptions in mitotic spindle formation, resulting in abnormal nuclear division. At later stages, such as trophozoites and schizonts, transient exposure to docetaxel inhibits mitosis by stabilizing microtubule structures associated with the mitotic apparatus, leading to the formation of defective spindles. Notably, this mitotic disruption does not appear to affect DNA replication, suggesting that the parasite lacks a regulatory checkpoint linking mitotic exit to proper spindle assembly, a feature typically observed in higher eukaryotic cells [81]. Although intracellular concentrations of docetaxel remain relatively low following treatment at the ring stage, these levels appear sufficient to disrupt spindle organization and interfere with normal cell division processes within the parasite [81]. It should be noted that docetaxel has been associated with cardiotoxicity, which limits its potential application as an antimalarial agent [82].

Docetaxel has also been evaluated against *Leishmania* spp., both as a single agent and in combination with established antileishmanial drugs. Docetaxel was assessed together with miltefosine and paromomycin, showing antileishmanial and immunomodulatory activity and supporting the potential use of taxane-based combination strategies for drug repurposing [74].

#### 3.2.3. Cisplatin

Cisplatin, a first-generation platinum-based anticancer drug, is widely used in oncology for the treatment of numerous solid malignancies.

Its antiparasitic potential has been evaluated in vivo models of visceral leishmaniasis. In a study conducted by Kaur and colleagues using BALB/c mice, cisplatin administration resulted in a significant reduction in parasite burden. The therapeutic effect was dose-dependent, with animals treated at 1 mg/kg body weight exhibiting a more pronounced decrease in parasite load compared to those receiving 0.5 mg/kg. This higher dosage was also associated with an enhanced delayed-type hypersensitivity (DTH) response and significant changes in biochemical parameters, including hepatic enzymes Serum Glutamic–Oxaloacetic Transaminase (SGOT, also known as AST or Aspartate Aminotransferase), Serum Glutamic–Pyruvic Transaminase (SGPT, also known as ALT or Alanine Aminotransferase), blood urea nitrogen (BUN), urea, creatinine, and phosphorus levels [83]. More recently, strategies aimed at improving the antiparasitic activity and selectivity of cisplatin have included the development of nanoformulations. Cisplatin conjugated to carbon nanotubes was evaluated in vitro against both promastigote and amastigote stages of *Leishmania major*. Particularly, cisplatin conjugated to multi-walled carbon nanotubes showed substantially greater activity against intracellular amastigotes than free cisplatin and meglumine antimoniate, while maintaining a favorable selectivity index [84].

The antiparasitic effects of cisplatin are not restricted to protozoan parasites. In *Schistosoma mansoni*, cisplatin showed activity against adult parasites both in vitro and in vivo. Treatment damaged the parasite tegument, reduced worm burden and viable egg production, and decreased the size and cellularity of egg-induced hepatic granulomas in infected mice [85]. Improvements in hepatic organization and biochemical indicators of liver function were also observed. Although interference with schistosome stem cell-like neoblasts has been proposed as a potential mechanism for antineoplastic drugs, a direct relationship between cisplatin activity and inhibition of parasite neoblasts remains to be demonstrated [85].

However, despite these promising antiparasitic effects, the potential clinical application of cisplatin as an antileishmanial agent is likely to be limited by its well-established toxicity profile. Moreover, cisplatin shares several dose-limiting adverse effects with pentavalent antimonials, including nephrotoxicity, peripheral neuropathy, severe nausea, vomiting, and myelosuppression, and cross-resistance between these compounds has also been reported, further limiting its potential therapeutic value for leishmaniasis [86].

#### 3.2.4. Doxorubicin

Doxorubicin is an anthracycline antitumor antibiotic and chemotherapeutic agent, widely used in the treatment of various cancers. Its primary mechanism of action involves intercalation into DNA, thereby disrupting DNA replication and transcription. The antiparasitic potential of doxorubicin has been evaluated both in vitro and in vivo against *Leishmania donovani*. In in vitro assays, the compound demonstrated strong activity against both promastigote and amastigote stages, with 50% effective dose (ED50) values of approximately 0.43 μM and 0.86 μM, respectively [87]. In murine models of infection, administration of doxorubicin at a dosage of 625 μg/kg body weight per day over four consecutive days resulted in up to a 95% reduction in splenic parasite burden. Notably, this dosage remains well below established toxicity thresholds, indicating a favorable therapeutic window [87]. These findings suggest that doxorubicin exhibits significant efficacy against visceral leishmaniasis and could be considered as a potential second-line treatment alongside agents such as amphotericin B and pentamidine.

Drug delivery strategies have also been explored to improve the selectivity of doxorubicin against intracellular *Leishmania donovani*. Macrophage-targeted chitosan-coated PLGA nanoparticles co-encapsulating doxorubicin and amphotericin B were developed for visceral leishmaniasis, exploiting the preferential uptake of particulate systems by macrophages to enhance delivery to parasite-containing cells [88]. Furthermore, enhanced therapeutic outcomes have been observed when doxorubicin is used in combination with amphotericin B. Delivery of both drugs to infected macrophages via mannose-functionalized nanomicelles resulted in a synergistic effect, improving antiparasitic activity against *Leishmania donovani*. This approach achieved low IC50 values (18.2 ng/mL for amphotericin B and 13.0 ng/mL for doxorubicin), highlighting the potential of targeted drug delivery systems to enhance treatment efficacy [89].

Doxorubicin has also demonstrated antiplasmodial activity in vitro. In erythrocytes infected with the chloroquine-resistant FCR-3 strain of *Plasmodium falciparum*, treatment with doxorubicin at 0.1 to 0.4 μM inhibited parasite development [90]. At this concentration, it also reduced parasite-associated erythrocyte lysis without inducing substantial lysis of uninfected erythrocytes. These findings suggest that the oxidative and DNA-targeting properties of doxorubicin may also interfere with blood-stage *Plasmodium falciparum*, although its well-established systemic toxicity remains an important limitation for therapeutic repurposing [90].

However, the antiparasitic activity of doxorubicin does not appear to extend uniformly across parasite taxa. In *Echinococcus multilocularis* metacestodes, short-term in vitro exposure to doxorubicin failed to produce sustained inhibition of parasite proliferation, and treated metacestodes subsequently developed parasite masses in vivo [91]. In the same experimental system, docetaxel showed markedly greater inhibitory activity. These findings emphasize that the antiparasitic potential of anticancer drugs cannot be extrapolated across parasite species and must be evaluated individually [91].

### 3.3. Antimicrobials Repurposed Against Parasites

#### 3.3.1. Azithromycin

Azithromycin is a second-generation macrolide characterized by several pharmacological advantages, including high oral bioavailability, rapid cellular uptake, extensive tissue distribution, and the convenience of once-daily dosing [92]. It has demonstrated broad-spectrum activity against pathogens responsible for respiratory infections, sexually transmitted diseases, and gastrointestinal infections [93,94,95].

This drug has also been recognized as a potent prophylactic agent, even against highly virulent strains of *Toxoplasma gondii*. Azithromycin is active against multiple developmental stages of the parasite, including tachyzoites, tissue cysts, and bradyzoites [92]. Additionally, alternative treatments such as artemisinin—derived from *Artemisia annua*—have been investigated for toxoplasmosis [96]. Traditionally, infusions of this plant have been used to treat malaria caused by *Plasmodium* species, another member of the Apicomplexa [97].

Studies evaluating the effectiveness of different treatments for preventing the vertical transmission of *Toxoplasma gondii*—including *Artemisia annua* infusion and spiramycin (SPFA)—in the rodent *Calomys callosus* as a model for congenital toxoplasmosis have provided valuable insights [98]. In this model, females were orally infected with 20 cysts of the parasite. Although parasites were detected in the placentas of azithromycin-treated animals, they were absent in the embryos. In contrast, embryos from females treated with *Artemisia annua* infusion exhibited signs of atrophy, while those treated with azithromycin remained free of detectable parasites. Conversely, tachyzoites were frequently observed in the livers of embryos from animals treated with SPFA, spiramycin, or *Artemisia annua*. Overall, azithromycin proved to be the most effective treatment, as it successfully prevented vertical transmission of *Toxoplasma gondii* in this experimental model [98].

Further studies have shown that *Artemisia annua* infusion can effectively control systemic infection with cyst-forming *Toxoplasma gondii* strains in C57BL/6 mice, offering a well-tolerated option due to its low toxicity and inhibitory effects on the parasite [96]. Moreover, in pregnant *Calomys callosus* treated with azithromycin, a reduction in parasite burden was observed in maternal brain tissue, and no parasites were detected in fetal ocular tissues, supporting its potential as an alternative therapeutic approach [98].

Recent evidence has confirmed the activity of azithromycin against *Toxoplasma gondii* in human trophoblast cells. In BeWo cells infected with two different parasite genotypes (TgChBrUD1 or TgChBrUD2), azithromycin treatment effectively controlled intracellular infection, providing additional evidence that its anti-*Toxoplasma* activity extends to cellular models relevant to the maternal–fetal interface [99]. Importantly, azithromycin has also been evaluated clinically for ocular toxoplasmosis. A recent retrospective comparative case series assessed azithromycin alone and in combination with pyrimethamine against conventional sulfadiazine–pyrimethamine therapy for *Toxoplasma*-related retinochoroiditis, providing contemporary clinical evidence for the potential use of azithromycin-containing regimens in toxoplasmosis [100].

For leishmaniasis, standard treatment traditionally relies on pentavalent antimonials such as sodium stibogluconate (Pentostam) or meglumine antimoniate [43]. Despite remaining first-line therapies worldwide, these drugs present significant limitations, including the need for parenteral administration, prolonged treatment regimens, and the risk of severe adverse effects related to toxicity [43]. In recent years, alternative treatments have been introduced, including paromomycin (aminosidine), lipid-based amphotericin B, oral miltefosine, and sitamaquine [101]. However, these options are often constrained by issues of cost, availability, and side effects. Notably, azithromycin has demonstrated in vitro activity against *Leishmania* species such as *L.* (*L.*) *amazonensis*, *L. braziliensis*, and *L. chagasi*, with effects observed in both promastigote and intracellular amastigote stages [43]. These findings suggest that azithromycin could represent a promising alternative in the treatment of leishmaniasis, particularly given the ongoing demand for therapies that are effective, safe, affordable, and easy to administer [43].

In addition, azithromycin has shown antimalarial activity against *Plasmodium falciparum*, both as a monotherapy and in combination with other antimalarial agents [102,103,104]. Its mechanism of action appears to involve direct effects on protozoan parasites such as *Toxoplasma gondii* and *Plasmodium* species, primarily through inhibition of protein synthesis within the apicoplast. This occurs via binding to the peptide exit tunnel of the 50S subunit of the parasite’s ribosome, thereby preventing peptide elongation [105,106,107,108,109]. More recently, a series of 17 azithromycin analogues have demonstrated significantly enhanced and rapid antiparasitic activity against *Plasmodium falciparum* and *Plasmodium knowlesi*, with up to a 38-fold increase in potency compared to the parent compound within 28 h of treatment. These compounds were effective during the blood-stage lifecycle, including ring stages, and retained activity even in parasites lacking an apicoplast, highlighting their potential as lead compounds for the development of improved antimalarial therapies [110]. Furthermore, evidence has strengthened the apicoplast as a relevant target of azithromycin in malaria parasites. In hepatic stages of *Plasmodium vivax* and *Plasmodium cynomolgi*, azithromycin disrupted apicoplast biogenesis in replicating parasites and affected dormant liver-stage forms. These findings extend the antiplasmodial activity of azithromycin beyond blood stages and provide recent mechanistic evidence supporting interference with apicoplast function as an important component of its antiparasitic activity [111].

#### 3.3.2. Nitrofurans

Nitrofurans are a class of synthetic antimicrobial compounds characterized by a nitrofuran ring and broad biological activity. Several members of this family have been investigated for potential antiparasitic applications, including nitrofurantoin against *Plasmodium* spp. and *Toxoplasma gondii* [112,113,114], nifuratel against *Leishmania donovani* [115], and furazolidone in intestinal protozoan infections [116,117], supporting the potential repurposing of this pharmacological class [112,113,115].

Nitrofurantoin is a bactericidal antibiotic belonging to the nitrofuran family. It was first approved in 1953 and has been used to treat acute and chronic lower urinary tract infections. However, its potential applications have expanded following evidence of activity against both drug-sensitive and drug-resistant strains of *Plasmodium falciparum*, specifically the *Pf* 3D7 and *Pf* Kelch13^R539T^ strains [36,112]. Shafi et al. (2023) cultured these two strains in vitro and evaluated the effects of nitrofurantoin alone and in combination with artemisinin, the current drug of choice for malaria treatment despite emerging resistance, at different concentrations [112]. They found that nitrofurantoin, at concentrations of 3.67 µM and 6.54 µM, inhibited parasite growth in both strains. Furthermore, it impaired development to the trophozoite stage and significantly increased the production of reactive oxygen species [112].

Notably, nitrofurantoin not only induced oxidative stress in the parasites but also compromised their antioxidant defense system. By reducing the enzymatic activity of glutathione reductase, it prevented the conversion of oxidized glutathione to its reduced form, thereby impairing the ability of *Plasmodium falciparum* to counteract oxidative damage [112].

In the same study, the authors also conducted an in vivo experiment using mice infected with *Plasmodium berghei*. Four treatment groups were evaluated: nitrofurantoin (40 mg/kg), artemisinin (60 mg/kg), and a combination of nitrofurantoin (20 mg/kg) with artemisinin (30 mg/kg), alongside the appropriate control group. The results showed a reduction in parasitemia and an increase in mouse survival time, with the most pronounced effects observed in the group receiving the combination therapy. These findings demonstrated a synergistic interaction between the two compounds, enhancing their antimalarial efficacy compared with either treatment administered alone [112].

Nitrofurantoin has also demonstrated activity against *Toxoplasma gondii* in both in vitro and in vivo experimental models. Evidence from a murine model of chronic toxoplasmosis showed that nitrofurantoin monotherapy reduced brain cyst burden by approximately 60%, whereas its combination with spiramycin produced a greater reduction of approximately 80%. These parasitological findings were accompanied by improvement in brain histopathological alterations, further supporting the potential activity of nitrofurantoin against chronic stages of *Toxoplasma gondii* infection [118]. More recently, nitrofurantoin showed significant antiparasitic activity during both acute and chronic experimental toxoplasmosis, with efficacy comparable to spiramycin. Notably, combined treatment with nitrofurantoin and spiramycin produced greater antiparasitic effects than either drug administered alone, suggesting that combination therapy may enhance antiparasitic efficacy. However, further studies are required to establish its safety, optimal dosing, and clinical applicability for toxoplasmosis [113,114].

Another nitrofuran that has demonstrated antiparasitic potential is nifuratel. Phenotypic screening of repurposing drug collections identified nifuratel as a promising candidate against *Leishmania* spp. Subsequent in vivo evaluation demonstrated that oral administration of nifuratel significantly reduced parasite burden in experimental visceral leishmaniasis, with reductions exceeding 80% under the evaluated treatment regimens [119].

A study conducted in 2023 demonstrated a synergistic effect between miltefosine and nifuratel against visceral leishmaniasis. This combination was effective against both axenic amastigotes and intracellular amastigotes within splenic macrophages from infected mice [115]. Furthermore, it significantly reduced the parasite burden in the thymus, liver, and bone marrow [115]. Although the mechanism underlying the interaction between these two compounds remains under investigation, the study demonstrated that treatment at a dose of 10 mg/kg/day for ten days was well tolerated and showed no significant safety concerns.

The combination of these two drugs may offer an opportunity to reduce the dose of miltefosine, a drug associated with teratogenic effects in mouse embryos and fetal toxicity. Although this possibility requires further investigation, the reported safety profile of nifuratel and the absence of major resistance concerns after decades of clinical use support further evaluation of this combination as a potential therapeutic strategy [115].

Other members of the nitrofuran family have also been evaluated against intestinal protozoa. Furazolidone, for example, has been investigated for its potential activity against *Cryptosporidium* spp. Early clinical observations in patients with AIDS-associated cryptosporidiosis reported partial or transient improvement of diarrhea following furazolidone treatment; however, *Cryptosporidium* oocysts frequently remained detectable in feces, indicating limited parasitological efficacy [116]. In veterinary medicine, a combination of metronidazole and furazolidone was evaluated during an outbreak of cryptosporidiosis in calves aged 1–2 months. Treatment resulted in clinical recovery, including resolution of diarrhea, as well as parasitological recovery based on the absence of detectable *Cryptosporidium* oocysts after treatment [117]. Although these observations suggest potential activity of furazolidone-containing regimens against cryptosporidiosis, the available evidence remains limited and does not establish the efficacy of furazolidone as monotherapy. Further controlled studies are therefore required to determine its specific contribution and potential for repurposing against *Cryptosporidium* spp.

Collectively, these findings indicate that the antiparasitic potential of nitrofurans extends beyond a single compound or parasite group. Nitrofurantoin, nifuratel, and furazolidone have shown activity in different experimental or clinical settings; however, the strength of evidence varies considerably among compounds and parasites. While recent experimental studies support further evaluation of nitrofurantoin and nifuratel for drug repurposing, the evidence for furazolidone against cryptosporidiosis remains preliminary and requires further validation.

### 3.4. Cardiovascular Drugs

#### Amiodarone

Amiodarone is a class III antiarrhythmic drug commonly used to regulate cardiac rhythm. In regions of Central and South America, co-infections involving *Trypanosoma cruzi* and *Leishmania* spp. are increasingly reported due to overlapping endemic areas. Despite this, therapeutic options for infections caused by flagellated kinetoplastids remain limited, and many currently available drugs are associated with significant toxicity. Although progress has been made in the development of new treatments for leishmaniasis, antimonial compounds are still widely used [120]. In contrast, treatment of Chagas disease remains challenging, particularly during the chronic phase, when the efficacy of currently available antiparasitic drugs is lower and therapeutic decisions depend on patient age, disease stage, and the presence of organ involvement. The identification of amiodarone as a nonconventional antiparasitic agent—particularly against *Leishmania*—has opened new avenues for therapeutic intervention in these diseases [120]. Recent studies have evaluated amiodarone in combination therapies for experimental cutaneous leishmaniasis. In hamsters infected with *Leishmania amazonensis*, amiodarone alone was unable to halt lesion development; however, its combination with pentavalent antimonial therapy improved treatment efficacy [121]. Amiodarone has also been evaluated against *Leishmania infantum*. Although the drug showed marked in vitro activity, particularly against intracellular amastigotes (IC_50_ = 0.5 μM), this effect did not translate into significant efficacy in an experimental hamster model of visceral leishmaniasis [122].

Chagas disease remains a major public health problem, with approximately 7 million people estimated to be infected with *Trypanosoma cruzi* worldwide, and chagasic cardiomyopathy representing one of its most severe clinical manifestations. Effective elimination of *Trypanosoma cruzi* is essential to halt disease progression; however, existing chemotherapeutic options are often limited by low efficacy and adverse effects. Amiodarone, widely prescribed for cardiac complications in Chagas patients, has demonstrated an unexpected direct antiparasitic effect against *Trypanosoma cruzi*, which is further enhanced when combined with ergosterol biosynthesis inhibitors such as posaconazole [123]. Amiodarone exerts its trypanocidal activity by disrupting intracellular calcium homeostasis. In *Trypanosoma cruzi*, calcium regulation depends primarily on the coordinated activity of the endoplasmic reticulum, the single mitochondrion, and the acidocalcisomes, the latter representing the major intracellular calcium storage organelles in trypanosomatids. Amiodarone collapses the mitochondrial membrane potential and simultaneously induces alkalinization of the acidocalcisomes, promoting the release of Ca^2+^ into the cytoplasm and ultimately leading to parasite death. In contrast, reservosomes are endocytic organelles specialized in protein and lipid storage and are not considered the primary regulators of calcium homeostasis in *Trypanosoma cruzi* [28,29,124].

Studies have shown that posaconazole also alters Ca^2+^ regulation and that its combination with amiodarone produces a synergistic effect. In addition, amiodarone inhibits sterol biosynthesis in *Trypanosoma cruzi*, an effect that is further potentiated by posaconazole [125]. The combined disruption of calcium homeostasis and inhibition of ergosterol synthesis—specifically at the level of oxidosqualene cyclase—likely underlies the strong antiproliferative activity observed with this drug combination. These findings provided the rationale for investigating amiodarone as a potentially dual-purpose agent in Chagas disease, combining its established antiarrhythmic activity with possible adjunct antiparasitic effects [126].

**Figure 3:**
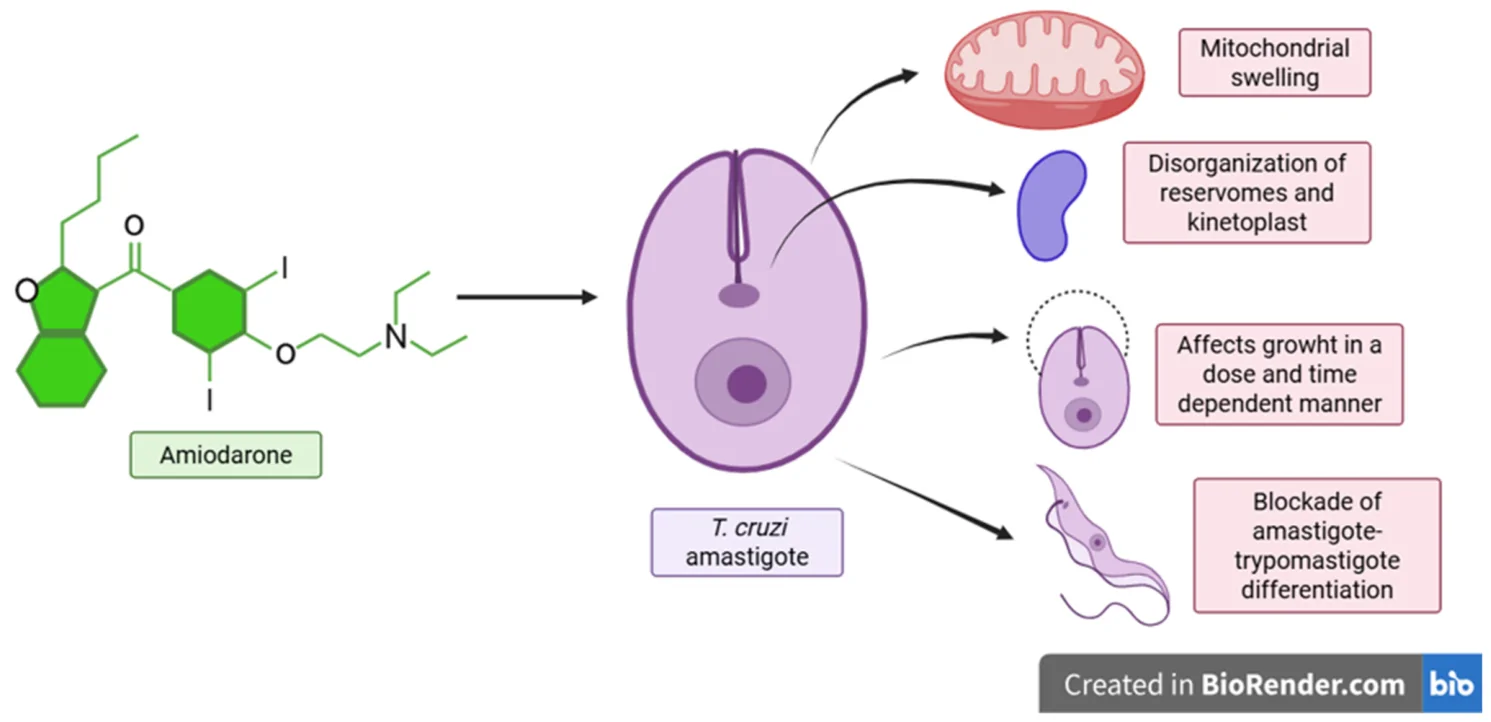
In vitro effect of amiodarone on *Trypanosoma cruzi* amastigotes in primary cardiac muscle cell cultures. The drug disrupts Ca^2+^ homeostasis in the parasite. Image created with BioRender.

In vitro models using infected primary cultures of cardiac muscle cells, amiodarone at concentrations ranging from 2.5 to 10 μM inhibited intracellular amastigote replication in a dose- and time-dependent manner. Ultrastructural analyses revealed marked alterations in the parasite, including mitochondrial swelling, disruption of reservosomes and kinetoplast organization, and inhibition of differentiation from amastigotes to trypomastigotes (Figure 3). These findings support the potential of amiodarone as a promising candidate for the development of new therapies targeting *Trypanosoma cruzi* [123].

More recently, the therapeutic potential of amiodarone has been evaluated in combination with low-dose benznidazole during acute experimental Chagas disease. The combined regimen produced a greater reduction in peak parasitemia than either monotherapy and was also associated with reduced cardiac inflammation, fewer electrocardiographic abnormalities, and improved gap-junction integrity [127]. Complementary in vitro studies using *Trypanosoma cruzi*-infected cardiac cells further showed that benznidazole–amiodarone combination therapy attenuated infection-associated cytoskeletal damage and promoted restoration of cardiac cellular organization [128]. The potential of this combination has subsequently been extended to chronic experimental Chagas disease. A 2024 preclinical study evaluated amiodarone together with low-dose benznidazole in chronically infected mice, providing further evidence for combination-based strategies aimed at simultaneously controlling parasite persistence and cardiac pathology [129].

However, more recent preclinical evidence has questioned the direct trypanocidal potential of amiodarone at clinically achievable exposures. In a comprehensive study using multiple *Trypanosoma cruzi* strains, host–cell systems, and bioluminescent murine models, amiodarone showed limited selectivity in vitro and failed to significantly reduce parasite burden during either acute or chronic infection at the highest tolerated dose [130]. These findings indicate that the direct antiparasitic efficacy of amiodarone remains controversial and may depend on parasite strain, drug exposure, experimental model, and whether the compound is administered alone or as part of a combination regimen [130].

Praziquantel remains the only widely available drug used for the treatment of schistosomiasis, underscoring the urgent need for novel anthelmintic agents. Screening of 46 cardiovascular drugs against *Schistosoma mansoni* identified amiodarone, along with telmisartan, propafenone, methyldopa, and doxazosin, as compounds capable of reducing parasite viability, with EC50 and EC90 values ranging from 8 to 50 μM. Among these, amiodarone exhibited the highest antischistosomal activity. In murine models of schistosomiasis, amiodarone treatment resulted in a significant reduction (>50%) in worm and egg burdens during early infection, whereas more modest effects (10–30% reduction) were observed in chronic stages. Notably, amiodarone demonstrated greater effectiveness in early infection compared to praziquantel [131].

Moreover, combination therapy with amiodarone and praziquantel (at doses of 200 or 400 mg/kg) in vivo produced a substantial reduction in worm burden, ranging from 60% to 70%. These findings suggest that combined regimens may enhance therapeutic outcomes and support the potential clinical application of amiodarone as part of combination therapy for schistosomiasis [132].

Collectively, the available evidence indicates that amiodarone exhibits antiparasitic activity against kinetoplastids and schistosomes through mechanisms involving disruption of Ca^2+^ homeostasis, mitochondrial function, and sterol metabolism. However, its efficacy varies substantially among parasite species and experimental models, and recent findings question whether direct trypanocidal activity can be achieved at clinically relevant exposures. Current evidence therefore appears to support the greatest potential for amiodarone as a partner in combination therapies rather than as a stand-alone antiparasitic drug.

## 4. Beyond Drug Repurposing

### Pharmacophores

Pharmacophores are the chemical structures responsible for a drug’s biological activity and its ability to establish effective interactions with its biological target. Examples include quinoline and imidazole, which are found in a variety of therapeutic agents. These structures can serve as templates for the development of new antiparasitic drugs, as well as for the redesign and repurposing of existing clinically approved medications [36].

Several studies conducted on pharmacophores such as quinoline have demonstrated their potential against diseases caused by protozoan parasites. Quinoline consists of a benzene ring fused to a pyridine ring and has already served as the structural basis for several antimalarial drugs, including quinine, quinidine, chloroquine, mefloquine, amodiaquine, primaquine, and bulaquine [133]. Recently, the ability of quinoline-based compounds to exhibit cross-efficacy against different protozoan parasites has been highlighted. This can be observed with amodiaquine and its analogue chloroquine, which have been shown to induce oxidative stress, mitochondrial dysfunction, and growth inhibition in *Leishmania infantum* and *Leishmania amazonensis*, respectively [36,134].

A similar pattern has been observed with tafenoquine against *Babesia* spp. and mefloquine against *Toxoplasma gondii*, both demonstrating significant antiprotozoal activity [36]. In the case of mefloquine, treatment significantly reduced mortality in infected animals (by up to 45%), decreased brain cyst burden (by up to 66.9%), and attenuated both acute and chronic inflammation induced by *Toxoplasma gondii* infection [36,135].

The potential of quinoline-related pharmacophores extends beyond malaria parasites and *Toxoplasma gondii*. Pharmacological optimization of the 8-nitroquinolin-2(1H)-one scaffold generated derivatives with potent antikinetoplastid activity. Particularly, a 6-bromo-substituted derivative showed nanomolar activity against *Trypanosoma brucei brucei* and submicromolar activity against intracellular *Trypanosoma cruzi* amastigotes [136].

In the case of imidazole, it has been proposed as a promising pharmacophore with potential applications against parasitic diseases. Several drugs containing this chemical structure, including omeprazole, triclabendazole, nitazoxanide, and rabeprazole, have been identified as candidates for repurposing and may exhibit antiparasitic activity [36].

Another important molecule is riboflavin, better known as vitamin B2, which was identified in one study as a pharmacophore active against *Schistosoma mansoni*. In this context, riboflavin targets the cysteine protease cathepsin B1, an enzyme expressed in the helminth’s digestive tract that plays a key role in host protein digestion. The researchers found that a concentration of 50 μM riboflavin was sufficient to inhibit the protease and resulted in parasite elimination in vitro within 24 h. Furthermore, they reported that oral administration of riboflavin at 100 mg/kg to infected mice reduced the parasite burden by 20% and decreased fecal egg counts by 80% [137].

These findings demonstrate that not only approved drugs, but also their core molecular scaffolds, may be repurposed. This opens a wide range of possibilities for future research and may lead to the development of novel therapeutic strategies based on the repositioning of bioactive pharmacophores.

## 5. Conclusions

The increasing emergence of antiparasitic drug resistance, together with the limited number of newly approved compounds and the high costs associated with conventional drug discovery, highlights the need for alternative therapeutic strategies. Drug repurposing has emerged as a practical approach for accelerating the identification of new antiparasitic therapies by exploiting the known pharmacological and safety profiles of approved drugs.

One of the principal conclusions of this review is that the host–parasite interface represents a valuable source of therapeutic opportunities. Advances in our understanding of neuroimmunoendocrine regulation, parasite signaling pathways, and host-derived hormonal influences have revealed molecular targets that can be exploited using compounds originally developed for oncology, endocrinology, infectious diseases, cardiovascular disorders, and other medical fields. The evidence reviewed here demonstrates that many of these drugs not only affect parasite viability directly but may also interfere with parasite development, reproduction, metabolism, or host immune responses.

Importantly, drug repurposing should not be viewed simply as the empirical screening of existing compounds. Rather, it should increasingly be guided by mechanistic knowledge of host–parasite interactions, parasite physiology, and molecular target identification. The integration of genomics, transcriptomics, structural biology, and computational drug discovery with experimental validation in vitro and in vivo will facilitate the rational selection of candidate compounds with greater specificity and reduced toxicity.

Although most of the evidence discussed in this review remains at the experimental stage, these studies collectively demonstrate that revisiting approved drugs provides a realistic opportunity to expand the antiparasitic therapeutic arsenal. Continued investigation of the host–parasite interface will not only improve our understanding of parasite biology but also accelerate the development of innovative, accessible, and more effective interventions for parasitic diseases affecting both humans and animals.

Ultimately, the future of antiparasitic drug repurposing will depend less on discovering new compounds than on improving our understanding of the molecular dialogue occurring at the host–parasite interface.

## Data Availability

The original contributions presented in this study are included in the article. Further inquiries can be directed to the corresponding author(s).

## References

[1] White A.C. (2003). Nitazoxanide: An Important Advance in Anti-Parasitic Therapy. Am. J. Trop. Med. Hyg..

[2] Gilles H.M., Hoffman P.S. (2002). Treatment of Intestinal Parasitic Infections: A Review of Nitazoxanide. Trends Parasitol..

[3] Kaushik A., Makarani N., Bharadava K., Gehlot J., Naik B.V., Singh A., Govil S., Kaushal R.S. (2025). Antiprotozoal Agents—Integration of Drug Discovery, Medicinal Chemistry, and Advanced Computational Approaches: An in-Depth Review. Microbe.

[4] Abboud P., Lemée V., Gargala G., Brasseur P., Ballet J.J., Borsa-Lebas F., Caren F., Favennec L. (2001). Successful Treatment of Metronidazole- and Albendazole-Resistant Giardiasis with Nitazoxanide in a Patient with Acquired Immunodeficiency Syndrome. Clin. Infect. Dis..

[5] De Vivo M., Masetti M., Bottegoni G., Cavalli A. (2016). Role of Molecular Dynamics and Related Methods in Drug Discovery. J. Med. Chem..

[6] Iasiukevich V.V., Zvantsov A.B. (1999). The Effect of Antihormones on the Susceptibility of *Anopheles sacharovi* Favre. Mosquitoes to the Causative Agent of Malaria *Plasmodium gallinaceum* Brumpt. Med. Parazitol..

[7] Pink R., Hudson A., Mouriès M.A., Bendig M. (2005). Opportunities and Challenges in Antiparasitic Drug Discovery. Nat. Rev. Drug Discov..

[8] Müller J., Hemphill A. (2016). Drug Target Identification in Protozoan Parasites. Expert Opin. Drug Discov..

[9] Hernández-Bello R., Escobedo G., Guzmán C., Ibarra-Coronado E.G., López-Griego L., Morales-Montor J. (2010). Immunoendocrine Host-Parasite Interactions during Helminth Infections: From the Basic Knowledge to Its Possible Therapeutic Applications: Review Article. Parasite Immunol..

[10] Aguilar-Díaz H., Nava-Castro K.E., Escobedo G., Domínguez-Ramírez L., García-Varela M., del Río-Araiza V.H., Palacios-Arreola M.I., Morales-Montor J. (2018). A Novel Progesterone Receptor Membrane Component (PGRMC) in the Human and Swine Parasite *Taenia solium*: Implications to the Host-Parasite Relationship. Parasit. Vectors.

[11] Woods D.J., Williams T.M. (2007). The Challenges of Developing Novel Antiparasitic Drugs. Invert. Neurosci..

[12] James C.E., Hudson A.L., Davey M.W. (2009). Drug Resistance Mechanisms in Helminths: Is It Survival of the Fittest?. Trends Parasitol..

[13] Kaplan R.M. (2020). Biology, Epidemiology, Diagnosis, and Management of Anthelmintic Resistance in Gastrointestinal Nematodes of Livestock. Vet. Clin. N. Am. Food Anim. Pract..

[14] Kaplan R.M. (2004). Drug Resistance in Nematodes of Veterinary Importance: A Status Report. Trends Parasitol..

[15] von Samson-Himmelstjerna G., Thompson R.A., Krücken J., Grant W., Bowman D.D., Schnyder M., Deplazes P. (2021). Spread of Anthelmintic Resistance in Intestinal Helminths of Dogs and Cats Is Currently Less Pronounced than in Ruminants and Horses—Yet It Is of Major Concern. Int. J. Parasitol. Drugs Drug Resist..

[16] Morales-Montor J., Chavarria A., De León M.A., Del Castillo L.I., Escobedo E.G., Sánchez E.N., Vargas J.A., Hernández-Flores M., Romo-González T., Larralde C. (2004). Host Gender in Parasitic Infections of Mammals: An Evaluation of the Female Host Supremacy Paradigm. J. Parasitol..

[17] Romano M.C., Jiménez P., Miranda-Brito C., Valdez R.A. (2015). Parasites and Steroid Hormones: Corticosteroid and Sex Steroid Synthesis, Their Role in the Parasite Physiology and Development. Front. Neurosci..

[18] Wesołowska A. (2022). Sex-the Most Underappreciated Variable in Research: Insights from Helminth-Infected Hosts. Vet. Res..

[19] vom Steeg L.G., Klein S.L. (2017). Sex Steroids Mediate Bidirectional Interactions Between Hosts and Microbes. Horm. Behav..

[20] Morales-Montor J., Colin-Oviedo Á., González G.M., Palma-Nicolás J.P., Sánchez-González A., Nava-Castro K.E., Domínguez-Ramírez L., García-Varela M., Del Río-Araiza V.H., Hernández-Bello R. (2022). Molecular Identification of a PGRMC-2 Receptor in Maturing Oocytes of the Zoonotic Nematode Parasite *Trichinella spiralis*. Vet. Parasitol..

[21] Escobedo G., Camacho-Arroyo I., Hernández-Hernández O.T., Ostoa-Saloma P., García-Varela M., Morales-Montor J. (2010). Progesterone Induces Scolex Evagination of the Human Parasite *Taenia solium*: Evolutionary Implications to the Host-Parasite Relationship. J. Biomed. Biotechnol..

[22] Scarpelli P.H., Pecenin M.F., Garcia C.R.S. (2021). Intracellular Ca^2+^ Signaling in Protozoan Parasites: An Overview with a Focus on Mitochondria. Int. J. Mol. Sci..

[23] Coelho N.C.C., Velásquez A.M.A., de Lima J.S., Ramos A.L.D., Cilli E.M., Graminha M.A.S. (2025). Calcium Homeostasis in Trypanosomatids: A Review of Molecular Targets and Inhibitors. Cell Calcium.

[24] Sharma M., Choudhury H., Roy R., Michaels S.A., Ojo K.K., Bansal A. (2021). CDPKs: The Critical Decoders of Calcium Signal at Various Stages of Malaria Parasite Development. Comput. Struct. Biotechnol. J..

[25] Lasonder E., More K., Singh S., Haidar M., Bertinetti D., Kennedy E.J., Herberg F.W., Holder A.A., Langsley G., Chitnis C.E. (2021). CAMP-Dependent Signaling Pathways as Potential Targets for Inhibition of *Plasmodium falciparum* Blood Stages. Front. Microbiol..

[26] Nawaratna S.S.K., You H., Jones M.K., McManus D.P., Gobert G.N. (2018). Calcium and Ca^2+^/Calmodulin-Dependent Kinase II as Targets for Helminth Parasite Control. Biochem. Soc. Trans..

[27] Docampo R., Moreno S.N. (2021). Calcium Signaling in Intracellular Protist Parasites. Curr. Opin. Microbiol..

[28] Docampo R., Huang G. (2022). New Insights into the Role of Acidocalcisomes in Trypanosomatids. J. Eukaryot. Microbiol..

[29] Benaim G., Paniz-Mondolfi A.E., Sordillo E.M., Martinez-Sotillo N. (2020). Disruption of Intracellular Calcium Homeostasis as a Therapeutic Target Against *Trypanosoma cruzi*. Front. Cell. Infect. Microbiol..

[30] Andrews K.T., Fisher G., Skinner-Adams T.S. (2014). Drug Repurposing and Human Parasitic Protozoan Diseases. Int. J. Parasitol. Drugs Drug Resist..

[31] Zumla A., Rao M., Wallis R.S., Kaufmann S.H.E., Rustomjee R., Mwaba P., Vilaplana C., Yeboah-Manu D., Chakaya J., Ippolito G. (2016). Host-Directed Therapies for Infectious Diseases: Current Status, Recent Progress, and Future Prospects. Lancet Infect. Dis..

[32] Wei L., Adderley J., Leroy D., Drewry D.H., Wilson D.W., Kaushansky A., Doerig C. (2021). Host-Directed Therapy, an Untapped Opportunity for Antimalarial Intervention. Cell Rep. Med..

[33] Scheiffer G., Domingues K.Z.A., Gorski D., Cobre A.D.F., Lazo R.E.L., Borba H.H.L., Ferreira L.M., Pontarolo R. (2024). In Silico Approaches Supporting Drug Repurposing for Leishmaniasis: A Scoping Review. EXCLI J..

[34] Charlton R.L., Rossi-Bergmann B., Denny P.W., Steel P.G. (2018). Repurposing as a Strategy for the Discovery of New Anti-Leishmanials: The-State-of-the-Art. Parasitology.

[35] Escobedo G., Roberts C.W., Carrero J.C., Morales-Montor J. (2005). Parasite Regulation by Host Hormones: An Old Mechanism of Host Exploitation?. Trends Parasitol..

[36] Hu S.S., Batool Z., Zheng X., Yang Y., Ullah A., Shen B. (2025). Exploration of Innovative Drug Repurposing Strategies for Combating Human Protozoan Diseases: Advances, Challenges, and Opportunities. J. Pharm. Anal..

[37] Alavi S.E., Ebrahimi Shahmabadi H. (2021). Anthelmintics for Drug Repurposing: Opportunities and Challenges. Saudi Pharm. J..

[38] Jordan V.C. (1999). Tamoxifen: Too Much of a Good Thing?. J. Clin. Oncol..

[39] Vargas-Villavicencio J.A., Larralde C., De León-Nava M.A., Escobedo G., Morales-Montor J. (2007). Tamoxifen Treatment Induces Protection in Murine Cysticercosis. J. Parasitol..

[40] Murray H.W., Berman J.D., Davies C.R., Saravia N.G. (2005). Advances in Leishmaniasis. Lancet.

[41] Zauli-Nascimento R.C., Miguel D.C., Yokoyama-Yasunaka J.K.U., Pereira L.I.A., Pelli De Oliveira M.A., Ribeiro-Dias F., Dorta M.L., Uliana S.R.B. (2010). In Vitro Sensitivity of *Leishmania* (*Viannia*) *braziliensis* and *Leishmania* (*Leishmania*) *amazonensis* Brazilian Isolates to Meglumine Antimoniate and Amphotericin B. Trop. Med. Int. Health.

[42] Berman J. (2003). Current Treatment Approaches to Leishmaniasis. Curr. Opin. Infect. Dis..

[43] De Oliveira-Silva F., De Morais-Teixeira E., Rabello A. (2008). Antileishmanial Activity of Azithromycin against *Leishmania* (*Leishmania*) *amazonensis*, *Leishmania* (*Viannia*) *braziliensis*, and *Leishmania* (*Leishmania*) *chagasi*. Am. J. Trop. Med. Hyg..

[44] Miguel D.C., Yokoyama-Yasunaka J.K.U., Andreoli W.K., Mortara R.A., Uliana S.R.B. (2007). Tamoxifen Is Effective against *Leishmania* and Induces a Rapid Alkalinization of Parasitophorous Vacuoles Harbouring *Leishmania* (*Leishmania*) *amazonensis* Amastigotes. J. Antimicrob. Chemother..

[45] Miguel D.C., Yokoyama-Yasunaka J.K.U., Uliana S.R.B. (2008). Tamoxifen Is Effective in the Treatment of *Leishmania amazonensis* Infections in Mice. PLoS Negl. Trop. Dis..

[46] Zewdie K.A., Hailu H.G., Ayza M.A., Tesfaye B.A. (2022). Antileishmanial Activity of Tamoxifen by Targeting Sphingolipid Metabolism: A Review. Clin. Pharmacol. Adv. Appl..

[47] Agostino V.S., Buerdsell M.L., Uliana S.R.B., Denny P.W., Coelho A.C., Steel P.G. (2024). Clemastine/Tamoxifen Hybrids as Easily Accessible Antileishmanial Drug Leads. Org. Biomol. Chem..

[48] Trinconi C.T., Miguel D.C., Silber A.M., Brown C., Mina J.G.M., Denny P.W., Heise N., Uliana S.R.B. (2018). Tamoxifen Inhibits the Biosynthesis of Inositolphosphorylceramide in *Leishmania*. Int. J. Parasitol. Drugs Drug Resist..

[49] Trinconi C.T., Reimão J.Q., Bonano V.I., Espada C.R., Miguel D.C., Yokoyama-Yasunaka J.K.U., Uliana S.R.B. (2018). Topical Tamoxifen in the Therapy of Cutaneous Leishmaniasis. Parasitology.

[50] Pennini S.N., de Oliveira Guerra J.A., Rebello P.F.B., Abtibol-Bernardino M.R., de Castro L.L., da Silva Balieiro A.A., de Oliveira Ferreira C., Noronha A.B., dos Santos da Silva C.G., Leturiondo A.L. (2023). Treatment of Cutaneous Leishmaniasis with a Sequential Scheme of Pentamidine and Tamoxifen in an Area with a Predominance of *Leishmania* (*Viannia*) *guyanensis*: A Randomised, Non-Inferiority Clinical Trial. Trop. Med. Int. Health.

[51] Silva T.C., Mengarda A.C., Silva B.C., Relvas-Lima T.S., Rodrigues V.C., Salvadori M.C., Teixeira F.S., Lopes A.F.S., Rando D.G.G., De Moraes J. (2021). New Evidence for Tamoxifen as an Antischistosomal Agent: In Vitro, in Vivo and Target Fishing Studies. Future Med. Chem..

[52] Landoni M., Piñero T., Soprano L.L., Garcia-Bournissen F., Fichera L., Esteva M.I., Duschak V.G., Couto A.S. (2019). Tamoxifen Acts on *Trypanosoma cruzi* Sphingolipid Pathway Triggering an Apoptotic Death Process. Biochem. Biophys. Res. Commun..

[53] Weinstock A., Gallego-Delgado J., Gomes C., Sherman J., Nikain C., Gonzalez S., Fisher E., Rodriguez A. (2019). Tamoxifen Activity against *Plasmodium* in Vitro and in Mice. Malar. J..

[54] Barakat A.M., El Fadaly H.A.M., Selem R.F., Madboli A.E.N.A., Abd El-Razik K.A., Hassan E.A., Alghamdi A.H., Elmahallawy E.K. (2022). Tamoxifen Increased Parasite Burden and Induced a Series of Histopathological and Immunohistochemical Changes During Chronic Toxoplasmosis in Experimentally Infected Mice. Front. Microbiol..

[55] Vargas-Villavicencio J.A., Larralde C., Morales-Montor J. (2006). Gonadectomy and Progesterone Treatment Induce Protection in Murine Cysticercosis. Parasite Immunol..

[56] Vargas-Villavicencio J.A., Larralde C., Morales-Montor J. (2008). Treatment with Dehydroepiandrosterone in Vivo and in Vitro Inhibits Reproduction, Growth and Viability of *Taenia crassiceps* Metacestodes. Int. J. Parasitol..

[57] Loria R.M., Padgett D.A. (1998). Control of the Immune Response by DHEA and Its Metabolites. Rinsho Byori.

[58] Carrero J.C., Cervantes C., Moreno-Mendoza N., Saavedra E., Morales-Montor J., Laclette J.P. (2006). Dehydroepiandrosterone Decreases While Cortisol Increases in Vitro Growth and Viability of *Entamoeba histolytica*. Microbes Infect..

[59] Fallon P.G., Richardson E.J., Jones F.M., Dunne D.W. (1998). Dehydroepiandrosterone Sulfate Treatment of Mice Modulates Infection with *Schistosoma mansoni*. Clin. Diagn. Lab. Immunol..

[60] Fulford A.J.C., Webster M., Ouma J.H., Kimani G., Dunne D.W., Fulford T. (1998). Puberty and Age-Related Changes in Susceptibility to Schistosome Infection. Parasitol. Today.

[61] Morales-Montor J., Newhouse E., Mohamed F., Baghdadi A., Damian R.T. (2001). Altered Levels of Hypothalamic-Pituitary-Adrenocortical Axis Hormones in Baboons and Mice during the Course of Infection with *Schistosoma mansoni*. J. Infect. Dis..

[62] Dos Santos C.D., Alonso Toldo M.P., Do Prado J.C. (2005). *Trypanosoma cruzi*: The Effects of Dehydroepiandrosterone (DHEA) Treatment during Experimental Infection. Acta Trop..

[63] Domínguez-Díaz L.R., Eugenia Ochoa M., Soto-Castro D., Farfán N., Morales-Chamorro M., Yépez-Mulia L., Pérez-Campos E., Santillan R., Moreno-Rodríguez A. (2021). In Vitro, Ex Vivo and in Vivo Short-Term Screening of DHEA Nitrate Derivatives Activity over *Trypanosoma cruzi* Ninoa and TH Strains from Oaxaca State, México. Bioorganic Med. Chem..

[64] Rasmussen K.R., Healey M.C. (1992). Dehydroepiandrosterone-Induced Reduction of *Cryptosporidium parvum* Infections in Aged Syrian Golden Hamsters. J. Parasitol..

[65] Muñiz-Hernández S., Luna-Nophal A., Gómez-De León C.T., Domínguez-Ramírez L., Patrón-Soberano O.A., Nava-Castro K.E., Ostoa-Saloma P., Morales-Montor J. (2021). Dehydroepiandrosterone Effect on *Toxoplasma gondii:* Molecular Mechanisms Associated to Parasite Death. Microorganisms.

[66] Escobedo G., Larralde C., Chavarria A., Cerbón M.A., Morales-Montor J. (2004). Molecular Mechanisms Involved in the Differential Effects of Sex Steroids on the Reproduction and Infectivity of *Taenia crassiceps*. J. Parasitol..

[67] Frincke J.M., Stickney D.R., Onizuka-Handa N., Garsd A., Reading C., Krudsood S., Wilairatana P., Looareesuwan S. (2007). Reduction of Parasite Levels in Patients with Uncomplicated Malaria by Treatment with HE2000. Am. J. Trop. Med. Hyg..

[68] Bojalil R., Terrazas L.I., Govezensky T., Sciutto E., Larralde C. (1993). Thymus-Related Cellular Immune Mechanisms in Sex-Associated Resistance to Experimental Murine Cysticercosis (*Taenia crassiceps*). J. Parasitol..

[69] Terrazas L.I., Bojalilt R., Govezensky T., Larralde C. (1998). Shift from an Early Protective TH1-Type Immune Response to a Late Permissive TH2- Type Response in Murine Cysticercosis (*Taenia crassiceps*). J. Parasitol..

[70] Morales-Montor J., Baig S., Mitchell R., Deway K., Hallal-Calleros C., Damian R.T. (2001). Immunoendocrine Interactions during Chronic Cysticercosis Determine Male Mouse Feminization: Role of IL-6. J. Immunol..

[71] Fitzpatrick S.L., Richards J.A.S. (1994). Identification of a Cyclic Adenosine 3′,5′-Monophosphate-Response Element in the Rat Aromatase Promoter That Is Required for Transcriptional Activation in Rat Granulosa Cells and R2C Leydig Cells. Mol. Endocrinol..

[72] Chavan H.D., Singh G., Dey C.S. (2007). Confocal Microscopic Investigation of Tubulin Distribution and Effect of Paclitaxel on Posttranslationally Modified Tubulins in Sodium Arsenite Resistant *Leishmania donovani*. Exp. Parasitol..

[73] Doherty T.M., Sher A., Vogel S.N. (1998). Paclitaxel (Taxol)-Induced Killing of *Leishmania major* in Murine Macrophages. Infect. Immun..

[74] Melcón-Fernández E., Balaña-Fouce R., García-Estrada C., Reguera R.M., Fernández-Rubio C., Cendón-Álvarez M., Pérez-Pertejo Y. (2026). Antileishmanial and Immunomodulatory Activity of Paclitaxel and Docetaxel Combined with Miltefosine and Paromomycin. Int. J. Mol. Sci..

[75] Pouvelle B., Farley P.J., Long C.A., Taraschi T.F. (1994). Taxol Arrests the Development of Blood-Stage *Plasmodium falciparum* in Vitro and *Plasmodium chabaudi adami* in Malaria-Infected Mice. J. Clin. Investig..

[76] Koka S., Bobbala D., Lang C., Boini K.M., Huber S.M., Lang F. (2009). Influence of Paclitaxel on Parasitemia and Survival of *Plasmodium berghei* Infected Mice. Cell. Physiol. Biochem..

[77] Estes R., Vogel N., Mack D., McLeod R. (1998). Paclitaxel Arrests Growth of Intracellular *Toxoplasma gondii*. Antimicrob. Agents Chemother..

[78] Tang Y., Zhang X., He X., Jiang T., Cao J., Yu X. (2026). Paclitaxel-Induced Tubulin Dysfunction Stalls Parasite Development: Synergistic Potential with Artemisinin against Resistant Strains. Microbiol. Spectr..

[79] Baum S.G., Wittner M., Nadler J.P., Horwitz S.B., Dennis J.E., Schiff P.B., Tanowitz H.B. (1981). Taxol, a Microtubule Stabilizing Agent, Blocks the Replication of *Trypanosoma Cruzi*. Proc. Natl. Acad. Sci. USA.

[80] Dantas A.P., Barbosa H.S., De Castro S.L. (2003). Biological and Ultrastructural Effects of the Anti-Microtubule Agent Taxol against *Trypanosoma cruzi*. J. Submicrosc. Cytol. Pathol..

[81] Sinou V., Boulard Y., Grellier P., Schrevel J. (1998). Host Cell and Malarial Targets for Docetaxel (Taxotere) during the Erythrocytic Development of *Plasmodium falciparum*. J. Eukaryot. Microbiol..

[82] Shimoyama M., Murata Y., Sumi K.I., Hamazoe R., Komuro I. (2001). Docetaxel Induced Cardiotoxicity. Heart.

[83] Kaur S., Sachdeva H., Dhuria S., Sharma M., Kaur T. (2010). Antileishmanial Effect of Cisplatin against Murine Visceral Leishmaniasis. Parasitol. Int..

[84] Akhtari J., Faridnia R., Kalani H., Bastani R., Fakhar M., Rezvan H., Beydokhti A.K. (2019). Potent in Vitro Antileishmanial Activity of a Nanoformulation of Cisplatin with Carbon Nanotubes against *Leishmania major*. J. Glob. Antimicrob. Resist..

[85] Eldeeb E., Fahmy S., Elbakry K., Hyder A. (2018). A Single Dose of the Antineoplastics Hydroxyurea or Cisplatin Has Praziquantel-like Effects on *Schistosoma mansoni* Worms and Host Mouse Liver. Biomed. Pharmacother..

[86] Naredi P., Heath D.D., Enns R.E., Howell S.B. (1994). Cross-Resistance between Cisplatin and Antimony in a Human Ovarian Carcinoma Cell Line. Cancer Res..

[87] Sett R., Basu N., Ghosh A.K., Das P.K. (1992). Potential of Doxorubicin as an Antileishmanial Agent. J. Parasitol..

[88] Singh P.K., Sah P., Meher J.G., Joshi S., Pawar V.K., Raval K., Singh Y., Sharma K., Kumar A., Dube A. (2016). Macrophage-Targeted Chitosan Anchored PLGA Nanoparticles Bearing Doxorubicin and Amphotericin B against Visceral Leishmaniasis. RSC Adv..

[89] Wei P., Ye Z., Cao S., Bai S., Seeberger P.H., Yin J., Hu J. (2020). Combination Therapy with Amphotericin B and Doxorubicin Encapsulated in Mannosylated Nanomicelles for Visceral Leishmaniasis. Colloids Surf. A Physicochem. Eng. Asp..

[90] Amari M.R., Wiraswati H.L., Fauziah N., Ma’Ruf I.F. (2022). Antimalarial Effect of Doxorubicin on Plasmodium Falciparum: An in Vitro Study in FCR-3 Strain. Biomed. Pharmacol. J..

[91] Huang X., Wiehr S., Wild A.M., Voßberg P., Hoffmann W., Grüner B., Köhler C., Soboslay P.T. (2018). The Effects of Taxanes, Vorinostat and Doxorubicin on Growth and Proliferation of *Echinococcus multilocularis* Metacestodes Assessed with Magnetic Resonance Imaging and Simultaneous Positron Emission Tomography. Oncotarget.

[92] Costa I.N., Angeloni M.B., Santana L.A., Barbosa B.F., Silva M.C.P., Rodrigues A.A., Rostkowsa C., Magalhães P.M., Pena J.D.O., Silva D.A.O. (2009). Azithromycin Inhibits Vertical Transmission of *Toxoplasma gondii* in *Calomys callosus* (Rodentia: Cricetidae). Placenta.

[93] Hook E.W., Stephens J., Ennis D.M. (1999). Azithromycin Compared with Penicillin G Benzathine for Treatment of Incubating Syphilis. Ann. Intern. Med..

[94] Togami K., Chono S., Morimoto K. (2013). Aerosol-Based Efficient Delivery of Azithromycin to Alveolar Macrophages for Treatment of Respiratory Infections. Pharm. Dev. Technol..

[95] Rakita R.M., Jacques-Palaz K., Murray B.E. (1994). Intracellular Activity of Azithromycin against Bacterial Enteric Pathogens. Antimicrob. Agents Chemother..

[96] de Oliveira T.C., Silva D.A.O., Rostkowska C., Béla S.R., Ferro E.A.V., Magalhães P.M., Mineo J.R. (2009). *Toxoplasma gondii:* Effects of *Artemisia annua* L. on Susceptibility to Infection in Experimental Models in Vitro and in Vivo. Exp. Parasitol..

[97] de Ridder S., van der Kooy F., Verpoorte R. (2008). *Artemisia annua* as a Self-Reliant Treatment for Malaria in Developing Countries. J. Ethnopharmacol..

[98] Lopes C.D., Silva N.M., Ferro E.A.V., Sousa R.A., Firmino M.L., Bernardes E.S., Roque-Barreira M.C., Pena J.D.O. (2009). Azithromycin Reduces Ocular Infection during Congenital Transmission of Toxoplasmosis in the *Calomys callosus* Model. J. Parasitol..

[99] Ribeiro M., Franco P.S., Lopes-Maria J.B., Angeloni M.B., Barbosa B.D.F., Gomes A.d.O., Castro A.S., da Silva R.J., de Oliveira F.C., Milian I.C.B. (2017). Azithromycin Treatment Is Able to Control the Infection by Two Genotypes of *Toxoplasma gondii* in Human Trophoblast BeWo Cells. Exp. Parasitol..

[100] Bohn M., Heeren T.F.C., de Silva I., Ajamil-Rodanes S., Petrushkin H. (2026). Azithromycin versus Azithromycin-Pyrimethamine and Sulfadiazine-Pyrimethamine for the Treatment of Ocular Toxoplasmosis: A Retrospective Comparative Case-Series. Eye.

[101] Matos A.P.S., Viçosa A.L., Ré M.I., Ricci-Júnior E., Holandino C. (2020). A Review of Current Treatments Strategies Based on Paromomycin for Leishmaniasis. J. Drug Deliv. Sci. Technol..

[102] Dunne M.W., Singh N., Shukla M., Valecha N., Bhattacharyya P.C., Dev V., Patel K., Mohapatra M.K., Lakhani J., Benner R. (2005). A Multicenter Study of Azithromycin, Alone and in Combination with Chloroquine, for the Treatment of Acute Uncomplicated *Plasmodium falciparum* Malaria in India. J. Infect. Dis..

[103] Miller R.S., Wongsrichanalai C., Buathong N., McDaniel P., Walsh D.S., Knirsch C., Ohrt C. (2006). Effective Treatment of Uncomplicated *Plasmodium falciparum* Malaria with Azithromycin-Quinine Combinations: A Randomized, Dose-Ranging Study. Am. J. Trop. Med. Hyg..

[104] Nakornchai S., Konthiang P. (2006). Activity of Azithromycin or Erythromycin in Combination with Antimalarial Drugs against Multidrug-Resistant *Plasmodium falciparum* in Vitro. Acta Trop..

[105] Cantin L., Chamberland S. (1993). In Vitro Evaluation of the Activities of Azithromycin Alone and Combined with Pyrimethamine against *Toxoplasma gondii*. Antimicrob. Agents Chemother..

[106] Biswas S. (2001). In-Vitro Antimalarial Activity of Azithromycin against Chloroquine Sensitive and Chloroquine Resistant *Plasmodium falciparum*. J. Postgrad. Med..

[107] Gingras B.A., Jensen J.B. (1992). Activity of Azithromycin (CP-62,993) and Erythromycin against Chloroquine-Sensitive and Chloroquine-Resistant Strains of *Plasmodium falciparum* in Vitro. Am. J. Trop. Med. Hyg..

[108] Ohrt C., Willingmyre G.D., Lee P., Knirsch C., Milhous W. (2002). Assessment of Azithromycin in Combination with Other Antimalarial Drugs against *Plasmodium falciparum* in Vitro. Antimicrob. Agents Chemother..

[109] McFadden G.I., Reith M.E., Munholland J., Lang-Unnasch N. (1996). Plastid in Human Parasites. Nature.

[110] Burns A.L., Sleebs B.E., Gancheva M., McLean K.T., Siddiqui G., Venter H., Beeson J.G., O’Handley R., Creek D.J., Ma S. (2022). Targeting Malaria Parasites with Novel Derivatives of Azithromycin. Front. Cell. Infect. Microbiol..

[111] Amanzougaghene N., Tajeri S., Franetich J.F., Ashraf K., Soulard V., Bigeard P., Guindo C.O., Bouillier C., Lemaitre J., Relouzat F. (2024). Azithromycin Disrupts Apicoplast Biogenesis in Replicating and Dormant Liver Stages of the Relapsing Malaria Parasites *Plasmodium vivax* and *Plasmodium cynomolgi*. Int. J. Antimicrob. Agents.

[112] Shafi S., Gupta S., Jain R., Shoaib R., Munjal A., Maurya P., Kumar P., Kalam Najmi A., Singh S. (2023). Tackling the Emerging Artemisinin-Resistant Malaria Parasite by Modulation of Defensive Oxido-Reductive Mechanism via Nitrofurantoin Repurposing. Biochem. Pharmacol..

[113] Yeo S.J., Jin C.M., Kim S.Y., Park H. (2016). In Vitro and in Vivo Effects of Nitrofurantoin on Experimental Toxoplasmosis. Korean J. Parasitol..

[114] Elkholy A., Wassef R., Alsaid O., Elawady M., Barakat A., Soror A., Kishik S. (2023). Evaluation of Mono and Combined Nitrofurantoin Therapy for Toxoplasmosis in Vivo Using Murine Model. Pathog. Glob. Health.

[115] Melcon-Fernandez E., Galli G., García-Estrada C., Balaña-Fouce R., Reguera R.M., Pérez-Pertejo Y. (2023). Miltefosine and Nifuratel Combination: A Promising Therapy for the Treatment of *Leishmania donovani* Visceral Leishmaniasis. Int. J. Mol. Sci..

[116] Epidemiologic Notes and Reports Cryptosporidiosis: Assessment of Chemotherapy of Males with Acquired Immune Deficiency Syndrome (AIDS). https://www.cdc.gov/mmwr/preview/mmwrhtml/00001187.htm.

[117] Randhawa S.S., Randhawa S.S., Zahid U.N., Singla L.D., Juyal P.D. (2012). Drug Combination Therapy in Control of Cryptosporidiosis in Ludhiana District of Punjab. J. Parasit. Dis..

[118] Abdallah K., Saleh M., Kishk S., Barakat A., Ali B., Elkholy A. (2022). A New Approach in Treatment of Chronic Murine Models of Toxoplasmosis Using Nitrofurantoin Antibiotic. Zagazig Univ. Med. J..

[119] Domínguez-Asenjo B., Gutiérrez-Corbo C., Álvarez-Bardón M., Pérez-Pertejo Y., Balaña-Fouce R., Reguera R.M. (2021). Ex Vivo Phenotypic Screening of Two Small Repurposing Drug Collections Identifies Nifuratel as a Potential New Treatment against Visceral and Cutaneous Leishmaniasis. ACS Infect. Dis..

[120] Paniz-Mondolfi A.E., Pérez-Álvarez A.M., Reyes-Jaimes O., Socorro G., Zerpa O., Slova D., Concepción J.L. (2008). Concurrent Chagas’ Disease and Borderline Disseminated Cutaneous Leishmaniasis: The Role of Amiodarone as an Antitrypanosomatidae Drug. Ther. Clin. Risk Manag..

[121] Anversa L., Salles Tiburcio M.G., Batista L.R., Cuba M.B., Nogueira Nascentes G.A., Martins T.Y., Richini Pereira V.B., Ruiz L.D.S., Dias da Silva V.J., Ramirez L.E. (2017). Amiodarone and Itraconazole Improve the Activity of Pentavalent Antimonial in the Treatment of Experimental Cutaneous Leishmaniasis. Int. J. Antimicrob. Agents.

[122] Pinto E.G., Tempone A.G. (2018). Activity of the Antiarrhythmic Drug Amiodarone against *Leishmania* (*L.*) *infantum*: An in Vitro and in Vivo Approach. J. Venom. Anim. Toxins Incl. Trop. Dis..

[123] Adesse D., Azzam E.M., De Meirelles M.N.L., Urbina J.A., Garzoni L.R. (2011). Amiodarone Inhibits *Trypanosoma cruzi* Infection and Promotes Cardiac Cell Recovery with Gap Junction and Cytoskeleton Reassembly in Vitro. Antimicrob. Agents Chemother..

[124] Docampo R. (2024). Advances in the Cellular Biology, Biochemistry, and Molecular Biology of Acidocalcisomes. Microbiol. Mol. Biol. Rev..

[125] Benaim G., Sanders J.M., Garcia-Marchán Y., Colina C., Lira R., Caldera A.R., Payares G., Sanoja C., Burgos J.M., Leon-Rossell A. (2006). Amiodarone Has Intrinsic Anti-*Trypanosoma cruzi* Activity and Acts Synergistically with Posaconazole. J. Med. Chem..

[126] Benaim G., Garcia C.R.S. (2011). Targeting Calcium Homeostasis as the Therapy of Chagas’ Disease and Leishmaniasis—A Review. Trop. Biomed..

[127] Barbosa J.M.C., Pedra Rezende Y., de Melo T.G., de Oliveira G., Cascabulho C.M., Pereira E.N.G.D.S., Daliry A., Salem K.S. (2022). Experimental Combination Therapy with Amiodarone and Low-Dose Benznidazole in a Mouse Model of *Trypanosoma cruzi* Acute Infection. Microbiol. Spectr..

[128] Barbosa J.M.C., Pedra-Rezende Y., Pereira L.D., de Melo T.G., Barbosa H.S., Lannes-Vieira J., de Castro S.L., Daliry A., Salomão K. (2022). Benznidazole and Amiodarone Combined Treatment Attenuates Cytoskeletal Damage in *Trypanosoma cruzi*-Infected Cardiac Cells. Front. Cell. Infect. Microbiol..

[129] Barbosa J.M.C., Pedra-Rezende Y., Mata-Santos H.A., Vilar-Pereira G., Melo T.G.D., Ramos I.P., Gibaldi D., Moreira O.C., Nunes D.F., Batista M.M. (2024). Preclinical Evaluation of Combined Therapy with Amiodarone and Low-Dose Benznidazole in a Mouse Model of Chronic *Trypanosoma cruzi* Infection. Biomed. Pharmacother..

[130] Francisco A.F., Chen G., Wang W., Sykes M.L., Escudié F., Scandale I., Olmo F., Shackleford D.M., Zulfiqar B., Kratz J.M. (2023). Preclinical Data Do Not Support the Use of Amiodarone or Dronedarone as Antiparasitic Drugs for Chagas Disease at the Approved Human Dosing Regimen. Front. Trop. Dis..

[131] Porto R., Mengarda A.C., Cajas R.A., Salvadori M.C., Teixeira F.S., Arcanjo D.D.R., Siyadatpanah A., Pereira M.D.L., Wilairatana P., de Moraes J. (2021). Antiparasitic Properties of Cardiovascular Agents against Human Intravascular Parasite *Schistosoma mansoni*. Pharmaceuticals.

[132] Cajas R.A., Santos S.S.B., Espírito-Santo M.C.C., Garedaghi Y., de Moraes J. (2024). In Vitro and in Vivo Efficacy of the Amiodarone and Praziquantel Combination against the Blood Fluke *Schistosoma mansoni*. Antimicrob. Agents Chemother..

[133] Yadav P., Shah K. (2021). Quinolines, a Perpetual, Multipurpose Scaffold in Medicinal Chemistry. Bioorg. Chem..

[134] Rossi N.R.D.L.P., Fialho S.N., Gouveia A.D.J., Ferreira A.S., da Silva M.A., Martinez L.D.N., Paula do Nascimento W.d.S., Gonzaga A., de Medeiros D.S.S., de Barros N.B. (2024). Quinine and Chloroquine: Potential Preclinical Candidates for the Treatment of Tegumentary Leishmaniasis. Acta Trop..

[135] El Sharazly B.M., Aboul Asaad I.A., Yassen N.A., El Maghraby G.M., Carter W.G., Mohamed D.A., Amer B.S., Ismail H.I.H. (2023). Mefloquine Loaded Niosomes as a Promising Approach for the Treatment of Acute and Chronic Toxoplasmosis. Acta Trop..

[136] Pedron J., Boudot C., Brossas J.Y., Pinault E., Bourgeade-Delmas S., Sournia-Saquet A., Boutet-Robinet E., Destere A., Tronnet A., Bergé J. (2020). New 8-Nitroquinolinone Derivative Displaying Submicromolar in Vitro Activities against Both *Trypanosoma brucei* and *cruzi*. ACS Med. Chem. Lett..

[137] Cogo R.M., Pavani T.F.A., Mengarda A.C.A., Cajas R.A., Teixeira T.R., Fukui-Silva L., Sun Y.U., Liu L.J., Amarasinghe D.K., Yoon M.C. (2024). Pharmacophore Virtual Screening Identifies Riboflavin as an Inhibitor of the Schistosome Cathepsin B1 Protease with Antiparasitic Activity. ACS Omega.

